# Developing and applying a training needs analysis tool for healthcare workers managing snakebite envenoming: A cross-sectional study in Eswatini

**DOI:** 10.1371/journal.pntd.0012778

**Published:** 2025-01-08

**Authors:** Jonathan Steinhorst, Clare Baker, Sara Padidar, Thea Litschka-Koen, Ezekiel Ngwenya, Lindelwa Mmema, Brent Thomas, Nondumiso Shongwe, Trevor Sithole, Mbongiseni Mathobela, Anna Trelfa, Nicholas R. Casewell, David G. Lalloo, Robert A. Harrison, Jonathan Pons, Ymkje Stienstra

**Affiliations:** 1 University of Groningen, University Medical Centre Groningen, Department of Internal Medicine/ Infectious Diseases, Groningen, The Netherlands; 2 Centre for Snakebite Research and Interventions, Liverpool School of Tropical Medicine, Liverpool, United Kingdom; 3 Mersey and West Lancashire Teaching Hospitals NHS Trust, United Kingdom; 4 Eswatini Snakebite Research and Intervention Centre, Simunye, Eswatini; 5 Eswatini Antivenom Foundation, Simunye, Eswatini; 6 Department of Biological Sciences, University of Eswatini, Kwaluseni, Eswatini; 7 Research Unit, Ministry of Health, Mbabane, Eswatini; 8 Ministry of Health, Neglected Tropical Disease Programme, Mbabane, Eswatini; Fundação de Medicina Tropical Doutor Heitor Vieira Dourado: Fundacao de Medicina Tropical Doutor Heitor Vieira Dourado, BRAZIL

## Abstract

A considerable number of patients present to hospitals in Eswatini each year following bites by venomous snakes. Effectively diagnosing and treating patients with snakebite envenoming requires healthcare workers to have a variety of generic and snakebite-specific medical skills. In several countries, however, healthcare workers have been found to have limited skills in managing snakebite patients. We used the Delphi method to adapt the Hennessy-Hicks training needs analysis questionnaire to the context of snakebite envenoming and subsequently used the adapted questionnaire to assess the self-perceived training needs of 90 healthcare workers from ten hospitals in Eswatini. Two-thirds (63%) of participants were nursing staff and one third (34%) medical doctors. Overall, 74% of healthcare workers had previously received training on snakebite. Although a training need was reported for all skills included in the survey, the extent of the training need varied between different skills and groups of healthcare workers. The highest average training need was registered in the domains ‘research and audit’ and ‘clinical tasks’ with the latter accounting for nine of the ten skills with the highest training need. Nurses reported a higher training need than doctors, especially for clinical tasks. Receiving snakebite training before as well as after obtaining the primary qualification was associated with the lowest average training need, particularly in clinical skills. Ninety-three percent of interviewed healthcare workers would welcome more frequent training opportunities on the clinical management of snakebite patients. This newly developed snakebite training needs analysis tool can aid in adapting training initiatives to a dynamic and evolving healthcare workforce and it is designed to be transferrable to snakebite endemic settings worldwide.

## Author summary

Snakebite envenoming is a life-threatening emergency occurring predominantly in low-and middle-income countries in the Global South. Studies involving healthcare workers in snakebite-endemic countries have indicated that recognizing and appropriately treating snakebite envenoming can pose a challenge to healthcare staff. Reducing the deaths and disabilities caused by snakebite envenoming, however, will require a strengthened, competent and skilled healthcare workforce. In this study, a panel of international snakebite experts designed a questionnaire based on skills essential to the clinical care of patients with snakebite envenoming and which can be employed in any region around the world to assess the snakebite-specific training needs of healthcare workers. The questionnaire was subsequently used in a sample of healthcare workers in Eswatini, a country in which snakebite envenoming is a public health concern. The measured training needs differed according to the skill in question, participants’ profession and the extent and context of previously received snakebite training. Healthcare workers who had attended specialized snakebite training in the past had a markedly reduced training need for clinical skills. This study provides a useful tool which can help tailor snakebite-specific clinical training initiatives to the needs of local and regional healthcare workers.

## Introduction

Bites by venomous snakes are a public health problem throughout much of South America, South Asia, and Sub-Saharan Africa and constitute challenging medical emergencies to deal with at the public health as well as the clinical level. Globally, the annual incidence of snakebite envenoming in humans ranges from 421 000–1 841 000, resulting in an estimated 20 000–94 000 deaths [1]. This makes snakebite envenoming the deadliest among the neglected tropical diseases [2]. Victims of SBE are complex patients to diagnose and treat, requiring a variety of skills on behalf of the involved healthcare staff. Local symptoms comprise of swelling and soft tissue destruction, such as blistering, bruising and necrosis of the skin [3–5]. Systemic symptoms include signs of neurotoxicity (e.g. ophthalmoplegia, flaccid/respiratory paralysis), haemorrhage and symptoms of internal organ failure [3–5]. Identifying the snake species implicated in a bite is often difficult, therefore a syndromic management approach is recommended, based on knowledge of the medically important snakes in a region and their characteristic syndromes of envenoming [4,5]. Facilities involved in the treatment of snakebite patients need to be equipped and prepared to i) institute general resuscitative measures such as circulatory and ventilatory support, ii) deal with local tissue complications, iii) administer antivenom and iv) to manage complications associated with treatment [4–6]. Antivenom is currently the only effective treatment available to neutralize circulating venom toxins [6], but antivenoms are scarce, have limited geographical snake coverage, can cause adverse reactions [7–9] and are generally unaffordable for the people they are meant to serve [10,11].

Multiple studies from different countries have shown that healthcare workers (HCWs) have insufficient knowledge when it comes to the assessment and management of snakebite patients [12]. For example, 40% of physicians and nurses in Laos were unaware of life-threatening syndromes of envenoming, such as coagulopathy and muscular paralysis, following bites by regionally endemic snakes [13]. Insufficient knowledge on snakes and syndromes of envenoming also hampers the clinical evaluation of snakebite patients, including the decision of when to give antivenom, the correct dosage [14,15] and how to recognize and treat adverse reactions [14,16]. In keeping with these findings, SBE is often either not part of medical/nursing training [13,17] or is insufficiently covered by the curriculum [18,19], and post-graduate training opportunities are still an exception [14]. Training on the management of patients with SBE has been shown to increase knowledge [13,17], to result in better patient care and reduce snakebite related mortality [20]. Improving and expanding training of HCWs in the field of snakebite envenoming will be pivotal to achieving success in the goal formulated by the World Health Organization (WHO) to halve snakebite-related morbidity and mortality by 2030 [21].

Eswatini is a land-locked country in southern Africa with a population of approximately 1.2 million [22]. In 2016, The World Bank estimated 36% of the population had an average daily income below the international poverty line of USD 2.15/day [23]. Approximately half of the labour force is employed in the agricultural and industrial sector [24] and more than half of the entire population are thought to practice subsistence agriculture [25]. Snakebites are a common occurrence in Eswatini, with an average of 466 snakebite cases recorded annually in the national snakebite registry [7]. Highly venomous snakes of medical importance in Eswatini include the Black mamba (*Dendroaspis polylepis*), the Snouted cobra (*Naja annulifera*), the Mozambique spitting cobra (*Naja mossambica*), the Rinkhals (*Hemachatus haemachatus*), the Puff adder (*Bitis arietans*), the Boomslang (*Dispholidus typus*) and the Vine snake (*Thelotornis capensis*) [26]. However, this list is not exhaustive, and a number of other venomous snakes exist as well. There are no medical schools in Eswatini, and snakebite training is partially covered in the emergency nursing courses of nursing training in Eswatini. Cognisant of the need, regular in-service training of HCWs and the public has been organized by the Eswatini Antivenom Foundation (EAF) in recent years [27] and in collaboration with the Ministry of Health (MoH). In addition, the treatment guidelines used in EAF training for many years were adopted by the MoH in 2021 [26]. In the present study, we aimed to develop a snakebite-specific training needs assessment tool (SB-TNA tool) and to identify HCWs’ perception of their training needs in snakebite management.

## Methods

### Ethics statement

Ethical approval was obtained from the Research Ethics Committee of the Liverpool School of Tropical Medicine, United Kingdom (Reference LSTM REF 21–006) and the Eswatini Health and Human Research Review Board, Eswatini (EHHRRB034/2021). Written informed consent was obtained from participating Delphi panel members and HCWs prior to enrolment in the study.

### Study design

A Delphi methodology was employed to design the SB-TNA tool in 2021, which was subsequently used in a sample of Eswatini HCWs in the form of a cross-sectional survey (May-June 2022).

### Preparation of the SB-TNA tool

The SB-TNA tool aimed to meet the following objectives: i) contain a broad and comprehensive overview of key skills needed by HCWs to effectively manage SBE, ii) to be easily understood by all tiers of HCWs, and iii) to conform to the specifications of the Hennessey-Hicks Training Needs Analysis Questionnaire (HH-TNA tool) to uphold the psychometric properties of the originally validated instrument. The tool is designed to assess participants’ perceptions of their training needs by assessing competencies relevant for their individual job roles. By focusing on competencies rather than knowledge, we intended to assess the self-perceived ability to translate knowledge into practice and to successfully achieve skills-related outcomes. Moreover, the knowledge required to effectively treat snakebites depends on regional factors, such as snake species, but competencies are universal qualities that can be applied to any context and healthcare system and thus ensure transferability of the SB-TNA tool.

To design the SB-TNA tool, we adapted the original HH-TNA tool template, introduced by Hennessy & Hicks at the University of Birmingham, and licensed to the WHO [28]. The HH-TNA tool comprises of three subsections: 1) a demographic section, 2) a skills rating section including 30 skills divided across five competency domains (research/audit, communication/ teamwork, clinical tasks, administration, management/supervisory tasks) relevant to the participants’ job, and 3) a final open-ended section for participants to suggest their needs for training, resources, and possible facility-and organizational improvements. The skills in the core section of the HH-TNA tool are rated in two different categories, A and B, which aim to measure the importance of the skill for one’s job role (A) and one’s personal performance on the same skill (B). A 7-point Likert scale is used for rating the categories. A score of 7 for each of the two respective categories indicates that a skill is very important (Category A) and is performed very well (Category B). Conversely, a score of 1 is used to rate skills that are deemed unimportant by the participant for their job role (on category A) and which are not well performed (on category B). The questionnaire can be extended by an additional two categories, C and D, using the same 7-point Likert scale. Category C serves as a measure of how much participants believe the skill could be improved through organizational change and category D captures in how far participants believe a skills-specific training could improve the skill in question. As the information gathered using categories C and D can be used to design training programs, we initially included these in the SB-TNA tool.

We first modified the demographic section of the HH-TNA tool to fit the context of clinical snakebite management in Eswatini. Next, the skills rating section was adjusted to accommodate snakebite-specific aspects of training, education and experience. Skills for the clinical management of snakebite patients were identified from the WHO clinical snakebite management guidelines [4,5]. Five researchers with expertise in public health and/or the clinical management of SBE from the Liverpool School of Tropical Medicine reviewed the preliminary list of skills. The draft version of the SB-TNA tool contained 60 skills, of which 30 were from the original (HH-TNA) and 30 added by the research team. This skills list was subsequently used as input for the first round of the Delphi panel. The ultimate aim of the Delphi procedure was to condense the list of 60 skills down to a shorter list of the most relevant skills to create a SB-TNA tool comprising core competencies of clinical snakebite management, and where modifications did not exceed a replacement of 8 and an addition of 10 skills compared to the original HH-TNA tool. These limits were applied to ensure the psychometric properties of the instrument were not compromised [28]. The third and last section of the questionnaire consisted of an open-ended question where participants were asked to indicate a need for training, resources, as well as facility-and organizational improvements they thought necessary. This third section was not changed. The final SB-TNA tool was pilot-tested in a small sample of Eswatini HCWs prior to its use in the larger sample of HCWs.

### The Delphi procedure and panellists

The absence of an evidence-based selection of skills relevant to the care of snakebite patients required us to rely on an expert-panel to develop the SB-TNA tool. We therefore employed the Delphi methodology as described by Woodcock et al. [29]. Panellists were selected based on their expertise in the clinical management of snakebite envenoming in low-and middle-income countries. Panellists were approached via e-mail, which included a participant information sheet with details of the study and a consent form that participants were asked to sign to confirm their participation. A snowballing technique was employed, whereby the initial group of potential panellists was encouraged to forward the Delphi invitation to peer experts of their choice. The draft version of the SB-TNA tool containing 60 skills was circulated to the Delphi panellists, who were asked to vote for each skill using the options ‘Keep’, ‘Remove’, ‘Modify’, or ‘Unsure’. Consensus was defined as an agreement of ≥ 75% among panellists for all Delphi rounds. Panellists were also provided with an open field for skills-specific suggestions. The CREDES checklist for conducting and reporting Delphi studies [30] was used as guidance (**S1 Appendix**).

### Sample population of HCWs

**Fig 1:**
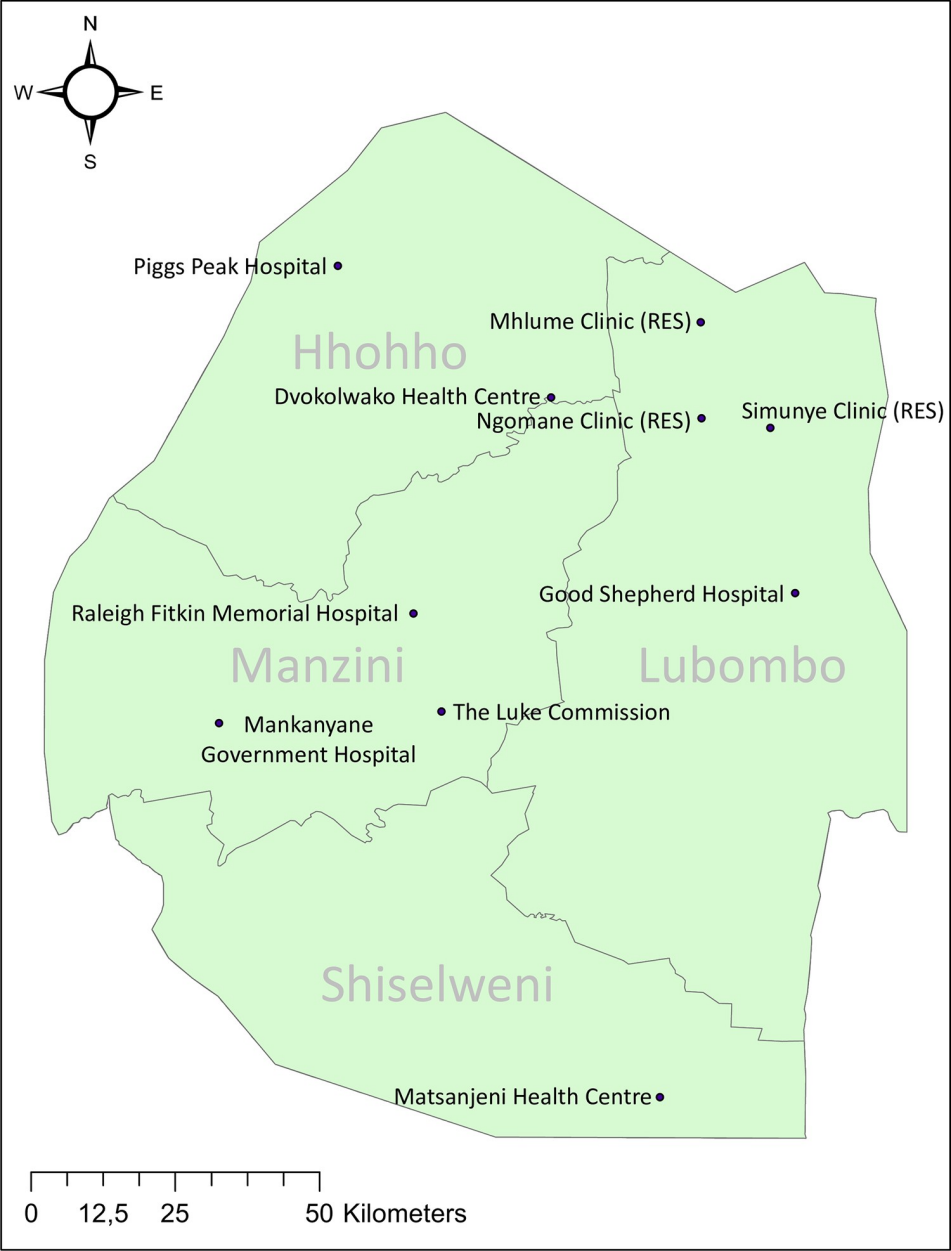
Map of Eswatini showing the location of the ten health facilities included in the healthcare worker survey. Based on geoBoundaries country dataset (CC BY 4.0 License) by Runfola et al. [31] and created using ArcGIS software version 10.8.1, ESRI Inc., Redlands, CA, U.S.A (modified to include names of regions and hospital locations). ‘RES’ denotes clinics operated by the Royal Eswatini Sugar Corporation.

HCWs from 10 health facilities with a combined healthcare workforce of 1133 HCW (1025 nurses, 108 doctors) and which routinely treat snakebite patients in Eswatini were included in the study (**Fig 1**). The ten health facilities, comprised of clinics, health centres and hospitals across the country, with differing types of available services (e.g. surgery, ICU, outpatient only), and are a representative sample of the 20 health facilities that can treat snakebite patients in Eswatini. Facilities were chosen to represent HCWs from the four administrative and the four agro-ecological regions (Highveld, Middleveld, Lowveld and Lubombo plateau) of the country (**Fig 1**). Two health centers (Dvokolowako Health Centre, Matsanjeni Health Centre) and four hospitals (Good Shepherd Hospital, Mankayane Government Hospital, Piggs Peak Government Hospital, Raleigh Fitkin Memorial Hospital) were government-operated, one of which is run jointly with a mission (Good Shepherd Hospital). One hospital (The Luke Commission) is run by a non-governmental organization and three clinics are operated by the Royal Eswatini Sugar Corporation (RES). Facilities were selected to represent healthcare provision with different types of service delivery, with hospitals having surgical specialties and intensive care (including ventilation) capacity whilst clinics provide only outpatient care. Consequently, the cross-section of healthcare facilities was representative of those that routinely manage snakebite patients.

### Administration of the SB-TNA tool

All HCWs involved in patient care and who could potentially be tasked with caring for snakebite patients were considered for inclusion in the study. HCWs not actively involved with bedside patient care were not included. HCWs were selected after consultation with the facility’s senior medical officers. In some facilities, senior medical officers delegated the task of selecting HCWs to heads of department, doctors or matrons to ensure that interviews did not interfere with the working schedule. Peer-to-peer recruitment of HCWs was common. The SB-TNA tool was administered in the form of interviews using the RedCap (Research Electronic Data Capture) mobile app [32] by two persons, JS and EN, who travelled to the involved health facilities in May and June 2022, shortly after the snakebite season (October-March) had ended. Interviews were conducted in English in a private setting, either in quiet outdoor areas in or around the hospital, or behind closed doors, e.g. conference or consultation rooms.

### Analysis

Demographic data was analysed using descriptive statistics. All statistical analyses were performed using IBM SPSS statistics version 29. To identify skills associated with a training need, the difference between importance and performance ratings was calculated. Ratings between groups of HCWs were analysed using independent samples t-test, Wilcoxon signed-rank test or Mann-Whitney-U-test as appropriate. Participant responses to part three of the questionnaire were reviewed and the most frequently mentioned resource gaps were reported. Suitable quotes of participants were selected to provide details of the context in which resource gaps and skill deficiencies were encountered, as well as on possible solutions offered by participants.

## Results

### Development of the SB-TNA tool

During the first round of the Delphi procedure, fifteen panellists reviewed and commented on the draft version of the SB-TNA tool that was shared in the initial e-mail. The professions of panellists ranged from being a medical doctor (n = 10), nurse (n = 3), public health specialist (n = 1), clinical research assistant (n = 1) to researcher (n = 7). Of note, each individual could select multiple professions. For instance, several medical doctors were researchers as well. Median time of involvement with the topic of snakebite was 15 years (1–45 years; min-max). Panellists had gathered their experience in snakebite in the following countries: Senegal, Ivory Coast, Ghana, Togo, Benin, Burkina Faso, Mali, Niger, Cameroon, Guinea, Uganda, Kenya, Eswatini, South Africa, India, Sri Lanka, Papua New Guinea, Brazil and Bolivia. In the first round, there was consensus amongst the panellists to keep 47 skills, of which 30 were from the original HH-TNA skills list. Panellists proposed an additional sixteen skills to the SB-TNA tool. General recommendations voiced by panel members were to avoid a ‘vertical approach’, in which skills applicable to a wide range of tasks and diseases are branded as ‘snakebite specific’. The underlying intention was to prevent the keyword of ‘snakebite’ from exaggerating the complexity of skills requiring similar capabilities and considerations as in other diseases and situations. Moreover, emphasis was placed on designing a SB-TNA tool that covers the breadth of facilities and treatment settings where snakebites are endemic. Following the first round, eight panellists convened in an online forum using Zoom [33] to discuss the content of the SB-TNA tool and the final skills to be included in the instrument. Panellists were encouraged to maintain anonymity through using pseudonyms, using the chat function, and disabling their webcams, but several panellists undertook neither of these steps and remained identifiable. Aside from wording and phrasing, participants were asked to anonymously cast a vote via private message to the lead researcher for the inclusion/exclusion of each skill and to discuss possible mergers of similar skills. The target was to arrive at a total of 40 skills to adhere to the modification limits set forth by the developers. Attrition of respondents during the Delphi panel discussion in round two and the need for the SB-TNA tool to conform to the specifications of the HH-TNA tool required a final vote on 13 remaining skills. This vote was conducted by way of an e-mail distributed to panellists, in which all modifications were approved. Overall, eight of the original 30 skills of the HH-TNA tool were modified and ten additional skills were added. Due to an error, the version of the SB-TNA tool used in the field did not contain one skill related to the indications for antibiotic treatment of snakebite patients. This mistake was only noticed once the SB-TNA tool had been administered. The SB-TNA tool as approved by the Delphi panel, including the question on the indications for antibiotics in snakebite, can be found in the **S2 Appendix.**

### Piloting of the SB-TNA tool

The SB-TNA tool was pilot tested with three HCWs (registered nurses). Interviews were perceived as lengthy, lasting ca. 45 minutes in total and especially the categories C and D were found to be repetitive and potentially compromised the overall quality of the data collected. Consequently, categories C and D were omitted, saving 15–20 minutes of interview time. Removal of categories C and D does not affect the validity of the SB-TNA tool but reduces the data available for choosing an effective method (training or organizational change) of delivering future training.

### Results of the training needs analysis in Eswatini HCWs

A total of ninety HCWs across the ten facilities were interviewed with a median of six HCWs (IQR 5–14) interviewed per facility. The sample comprised approximately 8% of the combined healthcare workforce working at the included facilities during the study year. Demographic characteristics of study participants are presented in **Table 1**. Forty-eight (53%) HCWs regularly worked in two or more departments. Twenty-nine participants (32%) reported that they had received training on snakebite management as part of their primary qualification. The median duration of this training was 3h (IQR 2-8h). Fifty-nine participants (66%) indicated to have received training on snakebite management in the context of continuous medical education, such as lectures, symposia, seminars or courses. Taken together, 67 HCWs (74%) had received training on snakebite at least once during their careers. The proportion of medical doctors who had received training after completion of their primary qualification was higher (n = 24, 77%) than that among nurses and nursing aides (n = 35, 61%). The majority of all HCWs (n = 47, 52%) reported receiving training by the EAF and eleven participants had received on-site training at their health facility. Two participants received their training abroad. When asked to recall the content of the training, 55 HCWs (93%) who had received such training (n = 59) stated that snakes, snake venoms and syndromes of envenoming were discussed. Instructions on topics such as diagnosis, treatment of snakebite envenoming and the management of adverse reactions to antivenom, were reportedly provided by 100% (n = 59), 98% (n = 58) and 95% (n = 56) of training sessions, respectively. HCWs pointed to multiple sources of guidance when managing a snakebite patient (**Table 1**). The Eswatini National Snakebite Management Guidelines [26] were explicitly mentioned by 65 participants and the proportion of HCWs who were aware of the guidelines (n = 51/65, 78%) was higher among previously trained individuals than among non-trained HCWs (n = 14/23, 61%).

**Table 1:** Demographic characteristics of HCWs (n = 90), experience in managing snakebite envenoming patients and mean training need.

	N (%)	Mean training need (SD)
**All healthcare workers**	90 (100)	1.85 (0.89)
**Gender** Female Male	43 (47.8) 47 (52.2)	2.07 (0.83) 1.65 (0.91)
**Age** 20-29 30-39 40-49 50+	18 (20.0) 44 (48.9) 16 (17.8) 12 (13.3)	2.03 (0.78) 1.87 (0.93) 1.78 (0.83) 1.61 (1.04)
**Professional role** Nurse assistant^1^ Registered nurse^2^ Medical doctor Other	6 (6.7) 51 (56.7) 31 (34.4) 2 (2.2)	2.51 (0.79) 1.99 (0.87) 1.38 (0.66) 3.49 (1.04)
**Years since obtaining primary qualification^3^ (Median; IQR)**	7 (4-13)	--
**Department^4^** Outpatient Internal Medicine Surgery Accident and Emergency Paediatrics Maternity Public Health Unit Other^5^	62 (68.9) 44 (48.9) 21 (23.3) 35 (38.9) 21 (23.3) 23 (25.6) 13 (14.4) 2 (2.2)	-- -- -- -- -- -- -- --
**Number of snakebite cases attended to in previous two years** 0 1-2 3-9 10-20 >20	6 (6.7) 12 (13.3) 35 (38.9) 17 (18.9) 20 (22.2)	2.15 (1.16) 2.08 (0.77) 1.92 (0.86) 1.58 (0.94) 1.74 (0.89)
**Sources of advice/guidance consulted by HCWs when managing snakebite patients** Senior staff in own facility Clinical guideline Peers in own facility Internet External specialist snakebite team Staff in another facility Textbook	76 (84.4) 72 (80) 50 (55.6) 50 (55.6) 47 (52.2) 12 (13.3) 6 (6.7)	-- -- -- -- -- -- --

^1^ Certificate of nursing assistant (2 years of training, focus on essential practical skills)

^2^ Diploma in general nursing (3 years) or a bachelor of nursing science and midwifery (4 years). Both include more theory and academic elements than the training for nursing assistant.

^3^ E.g. nursing or medical degree

^4^ Department where participant worked at the time at which the survey was performed. In case participants regularly worked in different departments, multiple departments could be selected per participant.

^5^ Includes occupational medicine, administration, psychiatry department, pharmacy

**Fig 2:**
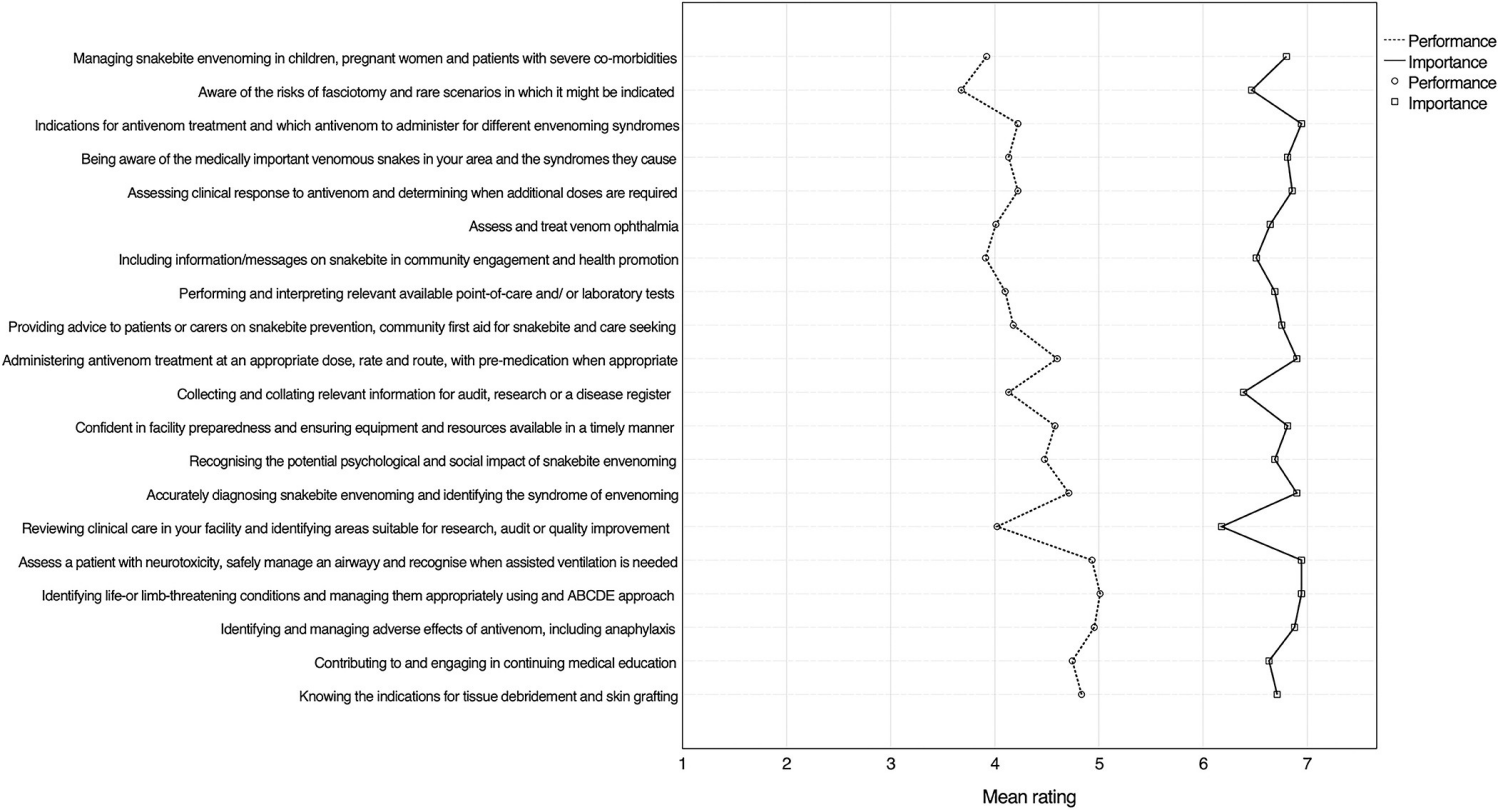
Graph showing the mean importance and performance rating of all HCWs (n = 90) for skills ranked 1–20 according to mean training need. Skills are ranked according to the difference between importance and performance ratings, which is depicted as the horizontal distance between the dashed and solid lines. Skills were abbreviated; the exact phrasing of skills can be found in the SB-TNA tool provided in the **S2 Appendix.** An overview of skills grouped according to domain is provided in the **S3 Appendix**.

**Fig 3:**
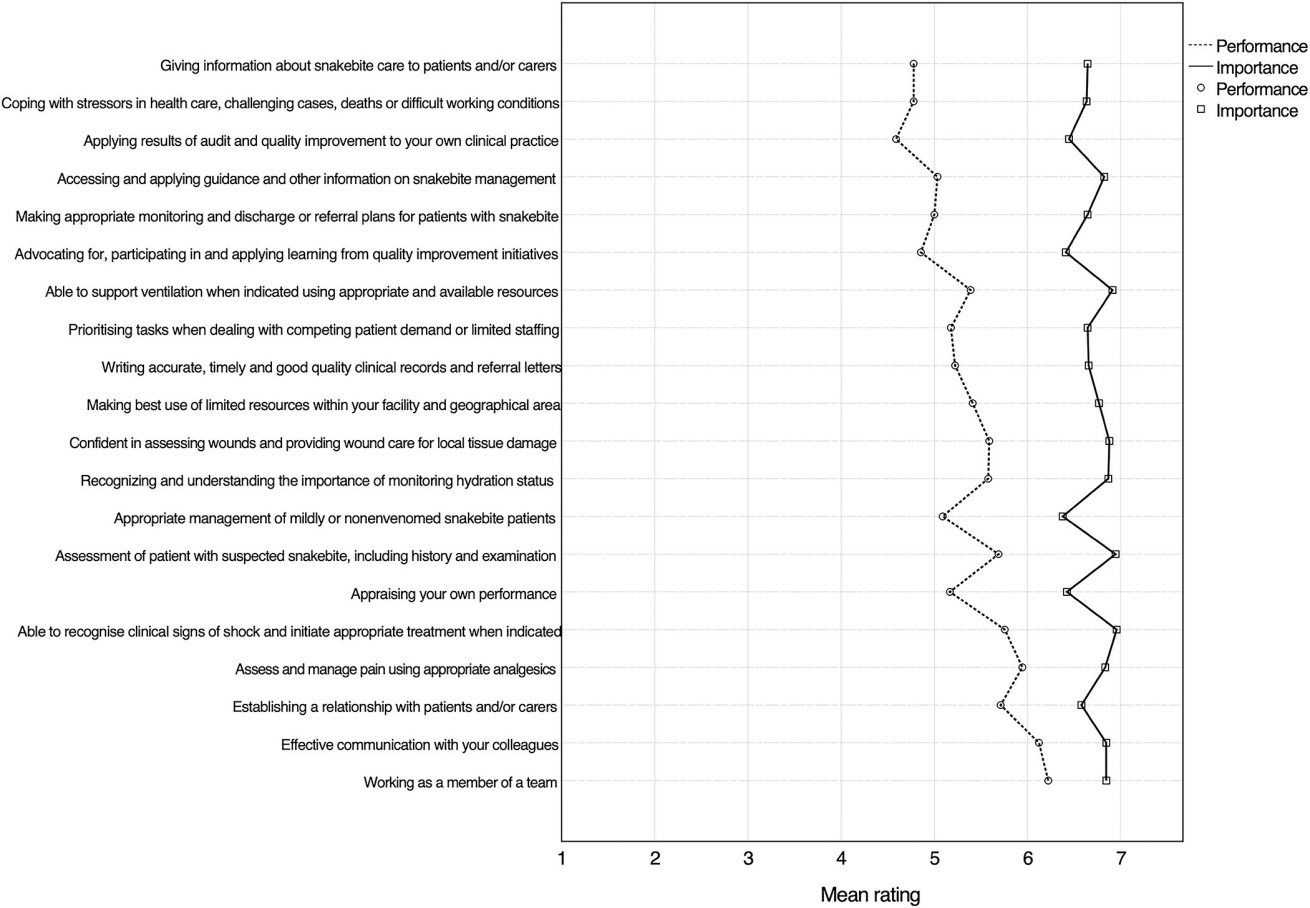
Graph showing the mean importance and performance rating of all HCWs (n = 90) for skills ranked 21–40 according to mean training need. Skills are ranked according to the difference between importance and performance ratings, which is depicted as the horizontal distance between the dashed and solid lines. Skills were abbreviated; the exact phrasing of skills can be found in the SB-TNA tool provided in the **S2 Appendix.** An overview of skills grouped according to domain is provided in the **S3 Appendix**.

An overview of HCWs’ self-perceived mean performance and importance scores for all forty skills is shown in **Figs 2** and **3**. HCWs on average rated all skills as being important to very important, with no single skill having a mean importance rating of less than six. Recognition and treatment of circulatory shock had the highest mean importance rating (6.96; SD 0.21). All ten skills with the highest mean importance rating belonged to the domain of clinical tasks. Conversely, when HCWs scored their performance of skills, clinical tasks made up seven of the ten lowest ranks. The lowest performance score was related to knowing indications for and risks of fasciotomy (mean performance rating 3.68; SD 1.98).

### HCWs perception of their training needs

**Fig 4:**
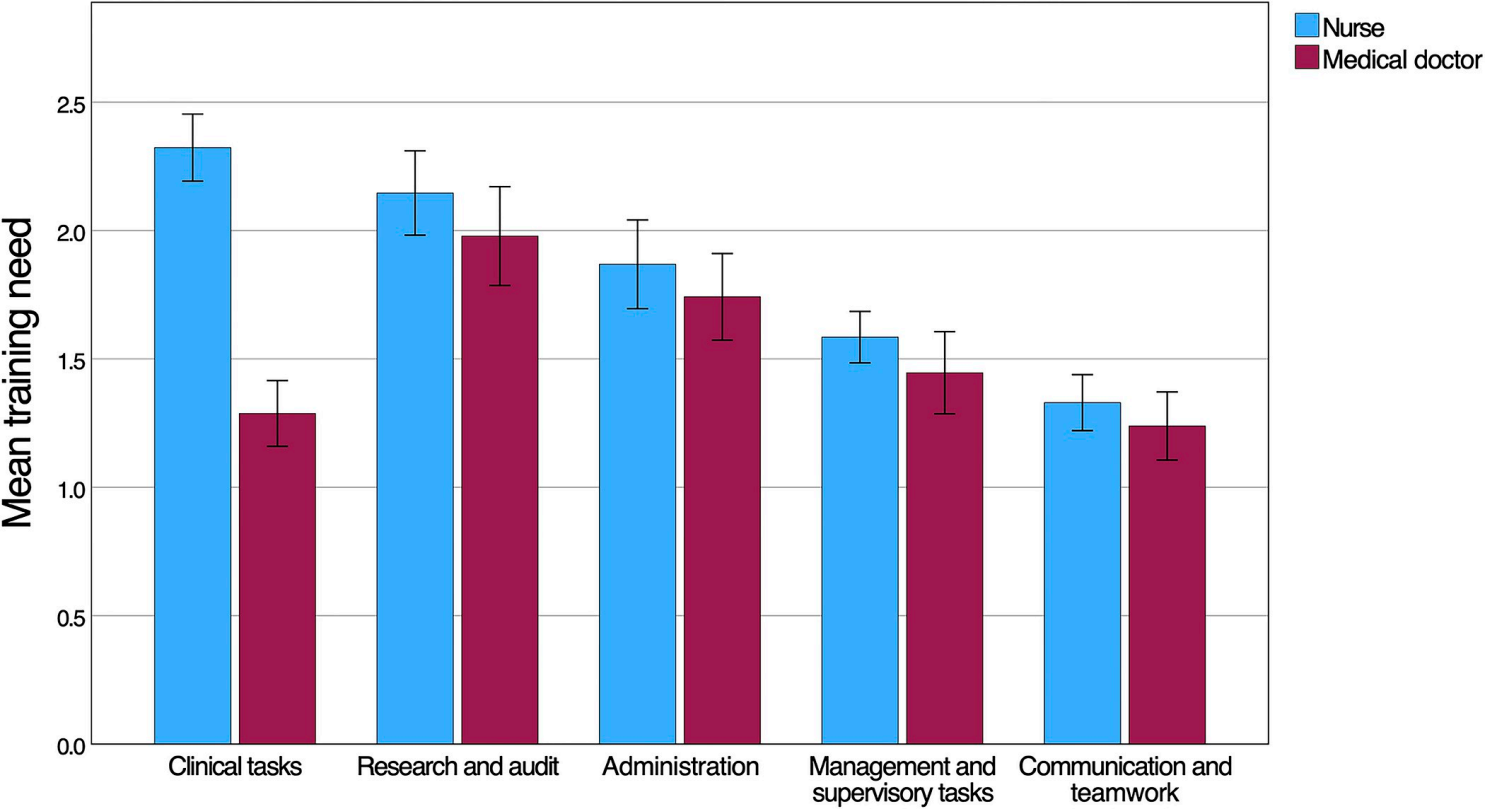
Mean training need for medical doctors (n = 31) and nurses (n = 57) shown per competency domain. The number of skills per domain are 24 (Clinical tasks), 3 (Research and audit), 2 (Administration), 6 (Management and supervisory tasks) and 5 (Communication and teamwork). Error bars represent standard errors.

**Fig 5:**
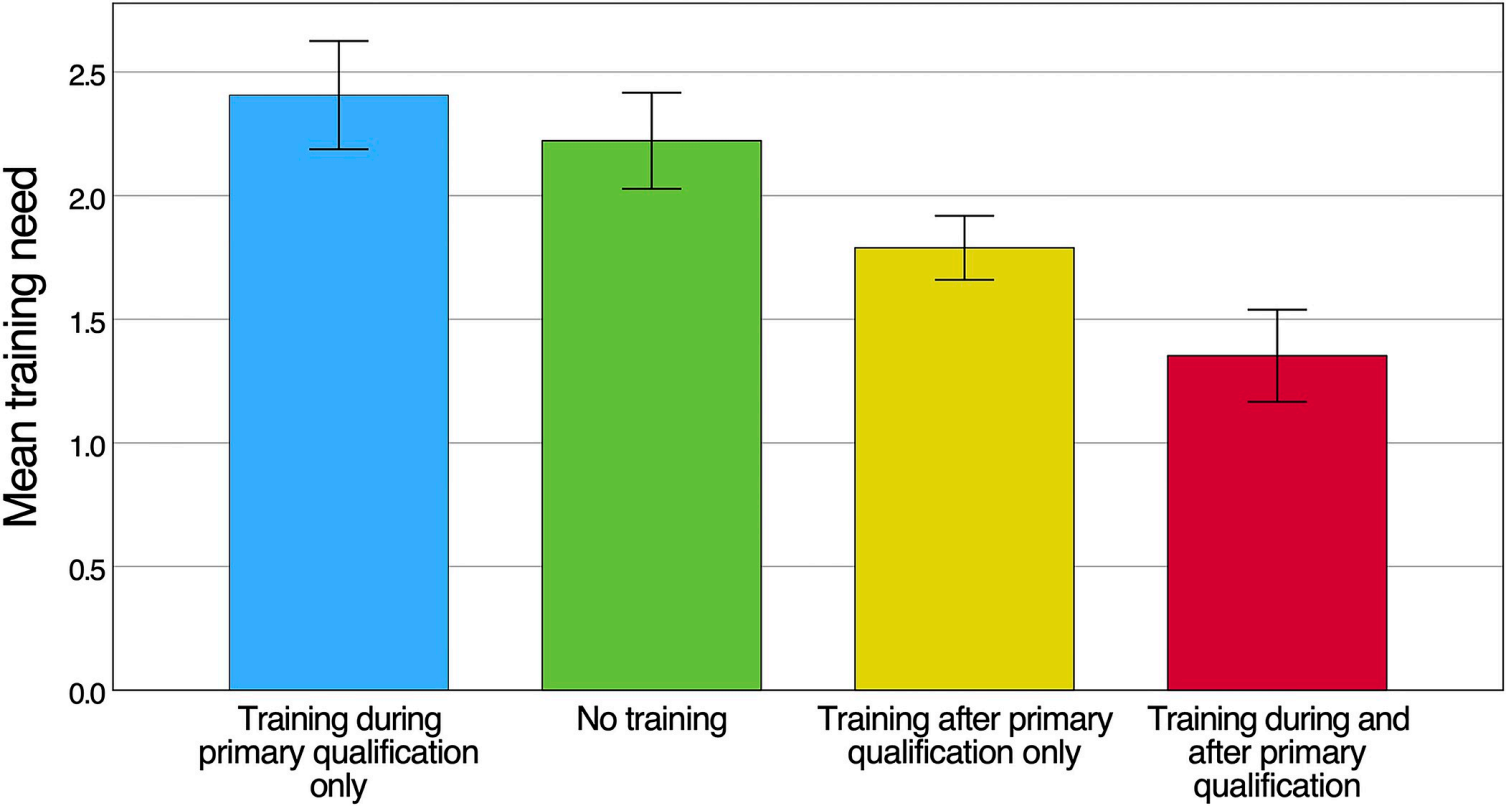
Training need of 90 HCWs who either did or did not receive different forms of training on snakebite management during different stages of their education/ careers. Group sizes are as follows: Training during primary qualification (n = 8), No training (n = 23), Training after primary qualification only (n = 38), and Training during and after primary qualification (n = 21). Error bars represent standard errors.

**Fig 6:**
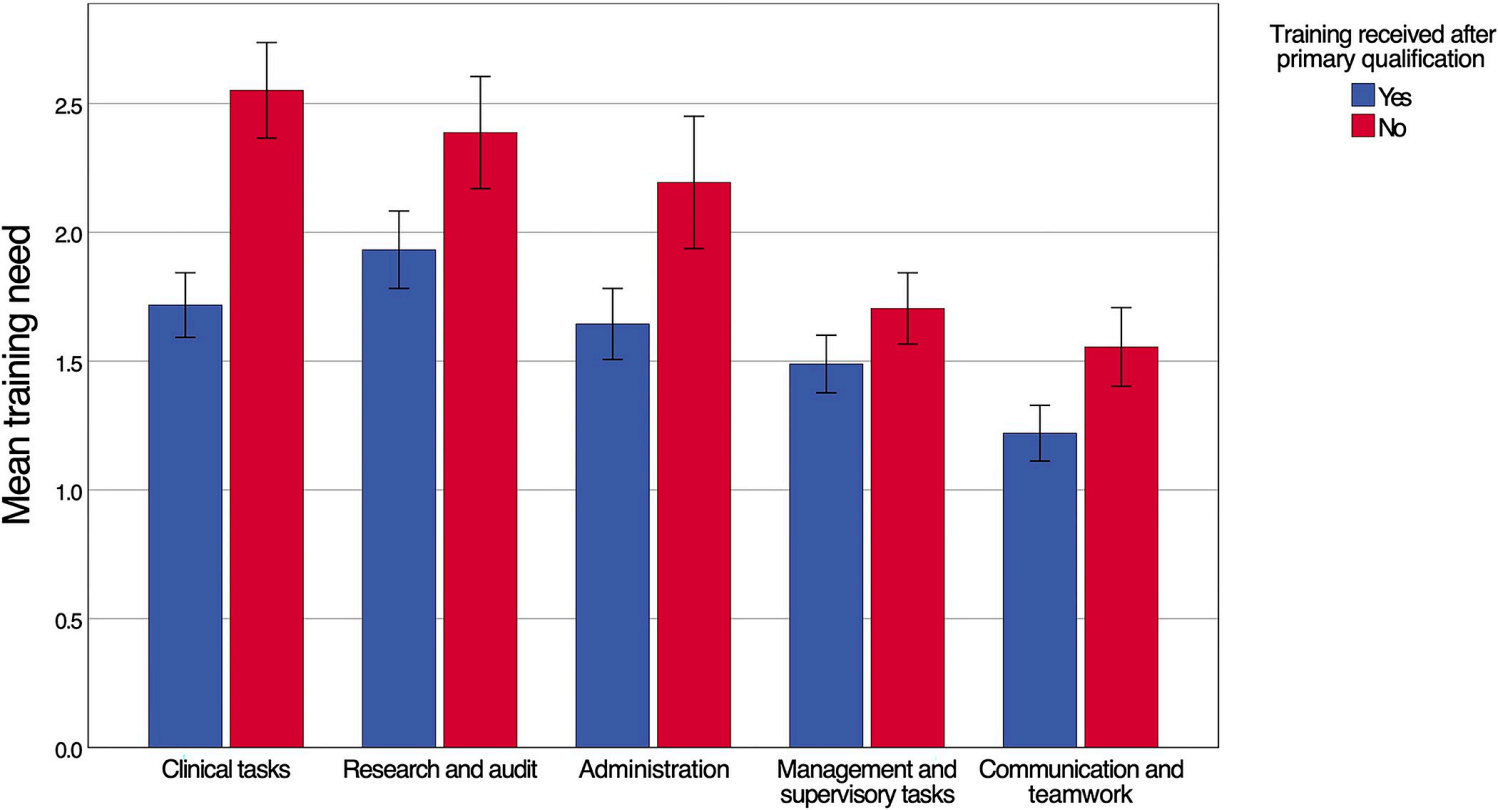
Mean training need for HCWs who received training on snakebite management after completing their primary qualification (n = 59) versus HCWs who had not (n = 31). The number of skills per domain are 24 (Clinical tasks), 3 (Research and audit), 2 (Administration), 6 (Management and supervisory tasks) and 5 (Communication and teamwork). Error bars represent standard errors.

Mean importance scores were higher than self-perceived performance scores for all forty skills (p<0.001), indicating a general training need. Overall, the highest training need (i.e. the difference between the importance and performance rating) was 2.88 (SD 1.89) and was related to understanding specific considerations when tending to children, pregnant women and patients with severe comorbidities affected by snakebite. The highest domain specific mean training need was observed for research and audit (2.09; SD 1.19), followed by clinical tasks (2.00; SD 1.06). Of the ten skills with the highest training need, nine belonged to the domain clinical tasks. A comparison of training needs between different competency domains is provided in **Fig 4**. Lower domain-specific average training needs were observed for administrative skills (1.83; SD 1.22), management and supervisory tasks (1.56; SD 0.83) and communication and teamwork (1.34; SD 0.85). HCWs reported higher training needs more commonly for snakebite-specific skills, while general tasks applicable to a wider context of patient care had lower training needs. Whilst this study was not powered to compare the different HCW cadres, it is worth noting that nurses (including nurse assistants) reported a higher training need (n = 57, mean 2.05; SD 0.87) compared to medical doctors (n = 31, mean 1.38; SD 0.66; p<0.001). This difference was most pronounced for clinical tasks (**Fig 4**). The higher self-perceived training need of nurses was driven primarily by clinical skills outside of the normal scope of work for nurses treating snakebite patients in Eswatini. Disaggregating the training need of HCWs according to the type of training received showed that the average training need was highest for HCWs who had only received training on snakebite while obtaining their primary qualification (n = 8, mean 2.41; SD 0.62) (**Fig 5**). In descending order, the next highest training need was observed for HCWs who had not received any form of snakebite training (n = 23, mean 2.22; SD 0.93), training only after obtaining the primary qualification (n = 38, mean 1.79; SD 0.80) and training during and after their primary qualification (n = 21, mean 1.35; SD 0.85). HCWs who did not receive training on snakebite after obtaining their primary qualification had significantly higher training needs across all five domains (p<0.001) with the largest gap related to clinical tasks (**Fig 6**). In general, training needs appeared to decrease with increasing age and experience of treating snakebite patients (**Table 1**). In a post hoc analysis, HCWs (n = 53) who were involved with the treatment of less than 10 snakebite patients in the preceding two years had a training need (mean 1.98; SD 0.87) that did not statistically significantly (P = 0.098) differ from the training need of HCWs who had managed ≥ 10 snakebite patients (n = 37; mean 1.67; SD 0.91).

### Improvements to snakebite care suggested by HCWs

When asked in the third section of the SB-TNA tool to provide ideas on how the medical treatment of snakebite patients could be improved, three main resource gaps were highlighted by HCWs. These included training, mentioned by 84 HCWs (93%), availability of antivenom (n = 61, 68%) and the need for more ventilators (n = 19, 21%).

There were a range of suggestions on how training sessions could be offered and implemented. The value of the annual national snakebite symposia and snakebite training offered by the EAF was underlined. Additionally, several HCWs highlighted the value of on-site trainings as these would enable more HCWs to attend. Many participants also said they would welcome trainings being offered more frequently.

*‘Bedside teaching*, *for e*.*g*. *now there is a snakebite patient in the ward*, *so going by the patient with the team and discussing*.*’* [JS 14, Registered nurse; Mean training need on ‘clinical tasks’ domain 2.67]

*‘And then training should be several days*, *so that you can immerse yourself in the subject*. *And seeing the snakes yourself would also help to remember and learn about snakebite*.*’* [EN 14, Registered nurse; Mean training need on ‘clinical tasks’ domain 3.17]

*‘Currently there is only annual EAF training in Simunye*. *Downside is that people who are at work cannot attend*. *Workshops by EAF should be held more frequently*. *More on-site training at facility*. *More guidelines and posters on treatment of snakebite would be helpful*.*’* [JS 51, Medical doctor; Mean training need on ‘clinical tasks’ domain 1.54]

HCWs highlighted a range of additional topics to be included in snakebite training, such as the pharmacology of antivenom and why it is not appropriate for all snakebites, how to deal with adverse antivenom reactions, wound care, the psychological implications following a bite event and how to educate communities and patients on snakes and snakebite. Trainings offered by EAF cover a comprehensive list of topics (**S4 Appendix**) and the same are included in the national snakebite treatment guidelines.

*‘How to recognize when antivenom needs to be given*. *Sometimes antivenom is given when not needed*, *then no more vials left*. *Management of anaphylaxis*.*’* [JS 31, Medical doctor; Mean training need on ‘clinical tasks’ domain 1.96]

Besides antivenom and ventilators, HCWs highlighted the need for additional intensive care unit beds and health products normally used for snakebite patients (e.g. dressings for wound management). Note, at the time this survey was administered, there was only one polyvalent antivenom registered for use in Eswatini. The manufacturer was experiencing significant supply challenges affecting the entire southern African region.

*‘Two years back a 5-year-old boy died because antivenom was unavailable*.*’* [JS 43, Medical doctor; Mean training need on ‘clinical tasks’ domain 0.96]

*‘Ventilatory support is lacking*, *only ventilators are in intensive care unit*. *No ventilator on the emergency room*.*’* [JS 25, Registered nurse; Mean training need on ‘clinical tasks’ domain 2.75]

*‘Special dressings for cytotoxic bites*, *for necrotic wounds*. *Those dressings had holes*, *do not remember the name*.*’* [JS 50, Nurse working in the pharmacy; Mean training need on ‘clinical tasks’ domain 4.33]

## Discussion

The global push to accurately appraise the burden of snakebite, develop novel diagnostic and therapeutic tools and to ultimately reduce the morbidity and mortality associated with SBE will largely rely on the skills and competencies of frontline HCWs [21]. We therefore developed a snakebite-specific training needs analysis tool based on a previously validated instrument and subsequently used this tool to assess HCWs’ perception of their snakebite-related training needs in Eswatini. The convened Delphi panel members had diverse professional backgrounds and eleven of the fifteen panellists brought extensive clinical experience to the discussion. The latter was clearly reflected in panellists’ consensus to exchange questions pertaining to specialized research skills (e.g. study design; statistical analysis) for questions very much related to essential emergency care of snakebite patients. Importantly, maintaining generic skills as such in the SB-TNA tool is highly relevant, since they are particularly sensitive to spill-over effects from non-snakebite related vertical and horizontal training initiatives [34] and should therefore be adequately accounted for.

In terms of age, this sample of HCWs is relatively young, but is consistent with the age distribution of the general healthcare workforce in Eswatini [35]. As doctors are far fewer in number than nurses and given their supervisory roles in patient care, doctors were sampled more proactively with the secondary intention of ensuring a sufficient group size for comparative analyses of training needs. The ratio of doctors to nurses is therefore not representative of the true ratio of doctors to nurses in Eswatini [35].

We found that Eswatini HCWs are experienced at caring for snakebite patients, with nearly half of all interviewed HCWs having tended to ≥10 snakebite patients in the two years prior to this study. However, HCWs did not perceive themselves to have the necessary skills to meet this demand, and reported training needs for all skills included in the SB-TNA tool. The highest average domain-specific training need was identified for research-related skills, although this category was only represented by three skills on the SB-TNA tool. Our questionnaire did not enquire to what extent research skills were part of participants’ primary training, but research skills are not part of the snakebite medical management training activities regularly offered by the EAF, which many HCWs had attended [36]. General health research capacity in Eswatini is 47 full-time equivalent (FTE) health researchers per 1 million inhabitants, which is below the average (62 FTE health researchers per 1 million inhabitants) for low and middle-income countries [37]. Improving research skills of HCWs at the clinical and public health level is integral to strengthening health research capacity and to addressing important evidence gaps in SBE [38]. There are ongoing initiatives such as the D43 Programme to strengthen public health research in Eswatini, however, these initiatives are primarily focused on HIV/TB [39]. Snakebite-specific research capacity is being addressed through the African Snakebite Alliance, in which Eswatini is a core member [40].

The self-perceived clinical training priorities of Eswatini HCWs are comparable to other countries, although the specific topics differed. For example, knowledge-based questionnaires in other countries identified knowledge gaps among HCWs in snake identification, correctly identifying syndromes of envenoming [13,15,19,41] and antivenom administration [14,17,19,42]. Whilst the domain of clinical tasks had a high training need, it was also the domain with the largest decrease in training need among participants who had received training after completion of their primary qualification. This confirms that the training sessions provided in the past, most of which were organized by the EAF, effectively addressed one of the most urgent training needs. The positive effect of training on snakebite-specific knowledge scores has been repeatedly shown [13,17,42–45]. Importantly, the benefits of regular in-service training can extend beyond the acquisition of task-specific skills through increasing HCW morale, motivation, job satisfaction and organizational commitment [46,47]. HCWs in our study, and in other countries [48–50], have repeatedly expressed their wish to receive regular training on managing snakebite patients. In the context of the HCW brain-drain affecting many snakebite endemic areas [51], the potential positive effect of training on workforce retention is an important consideration.

In designing future training initiatives, the question of ‘who needs to be trained?’ is equally important to the question of ‘what content needs to be trained?’. Selecting HCWs who have not yet received specialized instructions on snakebite management could maximize the utility of future training initiatives in Eswatini. Furthermore, HCWs not directly involved with the care of snakebite patients should be considered for training, to ensure these staff are equipped to handle snakebite patients when they are rotated between clinical departments or health facilities, as is the norm in low and middle-income countries. Potential trainees can further be selected based on profession. Compared to doctors, nurses’ training needs were particularly increased for skills that fall outside their standard responsibilities in the context of Eswatini healthcare institutions. This warrants further investigation to better understand nurses’ perception of the importance and their performance of these skills to their role and scope of work, and how these are aligned to the national snakebite treatment guidelines. The in-country training of nurses in Eswatini provides an opportunity for addressing this training need at the level of primary job training. Unexpectedly, HCWs who had never received any form of training on snakebite management had a similar training need as HCWs who had received snakebite training as part of their primary qualification. Since we did not contrast HCWs’ self-perceived training needs with their actual skills, it is not possible to say whether HCWs without training overestimated their competencies or did in fact have skills derived from sources other than formal training (e.g. experience). Ameade et al. [43] found that HCWs in Ghana who had never received training on snakebite perceived their knowledge to be higher than their actual knowledge. HCWs who are introduced to the topic of snakebite management during their primary professional training are potentially more sensitive to the scope of competencies this patient category requires, endowing them with a better ability to calibrate their self-assessment [52].

Knowledge gaps have been attributed to snakebite envenoming being absent or insufficiently incorporated into medical and nursing teaching curricula [13,17–19]. However, in our sample one third of participants stated that snakebite was discussed during their primary professional training and 74% had had snakebite management training in one form or another at some point during their careers. The proportion of HCWs previously trained in snakebite management was thus substantially higher than reported elsewhere [17,43,44,53]. The relatively small population and geographic size of Eswatini, the moderate number of nationwide health facilities, combined with the years-long advocacy of the EAF [36,54] has likely contributed to the extensive reach of specialized snakebite trainings. Targeted and specialized snakebite trainings in Eswatini have been shown to be successful in capacitating HCWs to manage snakebite envenoming. Building capacity in other countries will require resources and collaboration with local and international experts in the field of SBE. Nonetheless, the Delphi panel convened for this study also emphasized the importance of generic clinical skills in the treatment of snakebite patients. By identifying diagnostic and treatment principles that are common to snakebite envenoming and other diseases, training initiatives could reach more healthcare workers, while delivering a clinical skills-set with universal applicability [55].

### Strengths and limitations

Shortcomings in the development of the SB-TNA tool included attrition of respondents between the first and second round of the Delphi process and a lack of anonymity during the panel discussion, which could have led to a certain degree of response bias and ‘group think’ [56, p. 102]. For practical reasons we omitted categories C and D from the SB-TNA tool, which precluded us from assessing whether participants favoured organizational change or targeted training to improve specific skills. However, we found that answers provided in the open-ended section of the SB-TNA tool at least partially addressed these questions. The impact of the missing question on indications for antibiotics following SBE on the validity and reliability of the SB-TNA tool is likely minor, as this question belonged to the most represented domain on the SB-TNA tool, namely clinical tasks. Performing the survey in the form of an interview instead of allowing participants to rate skills in private might have introduced some level of bias into the results. In particular, the consistently high importance ratings across all skills could potentially be related to social desirability bias, although this trend also appeared in other studies employing the HH-TNA tool [57–59]. While statistical comparisons between different groups of HCWs were performed, these are largely exploratory and not based on a priori sample size calculations. Care was taken to select facilities that are representative of those treating patients with SBE across all of Eswatini, with HCWs recruited from 10 of the 20 facilities that have the infrastructure and human resources to treat snakebite. It must be emphasized that the SB-TNA tool measured self-perceived training needs and not actual competency of healthcare workers. Future studies could assess the degree to which HCW training translates into better clinical outcomes.

## Conclusion

HCWs in Eswatini regularly manage snakebite patients. Our modified SB-TNA tool detected differences in HCWs’ self-perceived training needs for various skills in snakebite management and between different subgroups of HCWs. Overall, training needs were most marked for research-related and clinical skills and nurses reported a higher training need than doctors. The SB-TNA tool is designed to be transferrable to other snakebite endemic settings around the world, where its utility and merit ought to be explored further. The extent to which snakebite training programs are effective at improving clinical outcomes remains a question to be addressed in the future.

## Supporting information

S1 Appendix Checklist for Conducting and Reporting Delphi Studies in palliative care (CREDES). (PDF)

S2 Appendix SB-TNA Tool Delphi Panel-approved version. (PDF)

S3 Appendix Index of SB-TNA Tool skills and domains. (XLSX)

S4 Appendix Content of healthcare worker training courses on the management of snakebite envenoming offered by the Eswatini Antivenom Foundation. (PDF)

## References

[1] Kasturiratne A, Wickremasinghe AR, De Silva N, Gunawardena NK, Pathmeswaran A, Premaratna R, et al. The global burden of snakebite: A literature analysis and modelling based on regional estimates of envenoming and deaths. PLoS Med. 2008;5: e218. doi: 10.1371/journal.pmed.0050218 18986210 PMC2577696

[2] Roberts NLS, Johnson EK, Zeng SM, Hamilton EB, Abdoli A, Alahdab F, et al. Global mortality of snakebite envenoming between 1990 and 2019. Nat Commun. 2022;13: 6160. doi: 10.1038/s41467-022-33627-9 36284094 PMC9596405

[3] Gutiérrez JM, Calvete JJ, Habib AG, Harrison RA, Williams DJ, Warrell DA. Snakebite envenoming. Nat Rev Dis Prim. 2017;3. doi: 10.1038/nrdp.2017.63 28980622

[4] The World Health Organization. Guidelines for the management of snake-bites. 2010 [cited 2024 Nov 17]. Available from: https://apps.who.int/iris/handle/10665/204464

[5] World Health Organization (WHO). Guidelines for the Prevention and Clinical Management of Snakebite in Africa. Brazzaville; 2010 [cited 2024 Nov 17]. Available from: https://www.who.int/publications-detail-redirect/9789290231684

[6] Warrell D. “Venomous and Poisonous Animals.” In: Farrar J, Hotez PJ, Junghanss T, Kang G, Lalloo D, White NJ, et al., editors. Manson’s Tropical Diseases (24th Edition). Elsevier Limited; 2023. pp. 1099–1023.

[7] Padidar S, Monadjem A, Litschka-koen T, Thomas B, Shongwe N, Baker C, et al. Snakebite epidemiology, outcomes and multi-cluster risk modelling in Eswatini. PLoS Negl Trop Dis. 2023;17: e0011732. doi: 10.1371/journal.pntd.0011732 37948462 PMC10664941

[8] Stone SF, Isbister GK, Shahmy S, Mohamed F, Abeysinghe C, Karunathilake H, et al. Immune Response to Snake Envenoming and Treatment with Antivenom; Complement Activation, Cytokine Production and Mast Cell Degranulation. PLoS Negl Trop Dis. 2013;7: e2326. doi: 10.1371/journal.pntd.0002326 23936562 PMC3723557

[9] Pattinson JP, Kong VY, Bruce JL, Oosthuizen G V., Bekker W, Laing GL, et al. Defining the need for surgical intervention following a snakebite still relies heavily on clinical assessment: The experience in Pietermaritzburg, South Africa. South African Med J. 2017;107: 1082–1085. doi: 10.7196/SAMJ.2017.v107i12.12628 29262961

[10] Casewell NR, Jackson TNW, Laustsen AH, Sunagar K. Causes and Consequences of Snake Venom Variation. Trends Pharmacol Sci. 2020;41: 570–581. doi: 10.1016/j.tips.2020.05.006 32564899 PMC7116101

[11] Brown NI. Consequences of neglect: Analysis of the sub-saharan african snake antivenom market and the global context. PLoS Negl Trop Dis. 2012;6: e1670. doi: 10.1371/journal.pntd.0001670 22679521 PMC3367979

[12] Michael GC, Bala AA, Mohammed M. Snakebite knowledge assessment and training of healthcare professionals in Asia, Africa, and the Middle East: A review. Toxicon X. 2022;16: 100142. doi: 10.1016/j.toxcx.2022.100142 36438018 PMC9692023

[13] Inthanomchanh V, Reyer JA, Blessmen J, Phrasisombath K, Yamamoto E, Hamajima N. Assessment of knowledge about snakebite management amongst healthcare providers in the provincial and two district hospitals in Savannakhet Province, Lao PDR. Nagoya J Med Sci. 2017;79: 299–311. doi: 10.18999/nagjms.79.3.299 28878435 PMC5577016

[14] Schurer JM, Hirwa EM, Masimbi O, Nduwayezu R. Knowledge, attitudes, and practices: a quantitative assessment of hospital physicians and medical interns treating snakebite envenomation in Rwanda. Trans R Soc Trop Med Hyg. 2022;116: 645–654. doi: 10.1093/trstmh/trab187 35016224

[15] Michael GC, Grema BA, Aliyu I, Alhaji MA, Lawal TO, Ibrahim H, et al. Knowledge of venomous snakes, snakebite first aid, treatment, and prevention among clinicians in northern Nigeria: a cross-sectional multicentre study. Trans R Soc Trop Med Hyg. 2018;112: 47–56. doi: 10.1093/trstmh/try028 29617989

[16] Fung HTJ, Lam SKT, Lam KK, Kam CW, Simpson ID. A Survey of Snakebite Management Knowledge Amongst Select Physicians in Hong Kong and the Implications for Snakebite Training. Wilderness Environ Med. 2009;20: 364–372. doi: 10.1580/1080-6032-020.004.0364 20030446

[17] Taieb F, Dub T, Madec Y, Tondeur L, Chippaux JP, Lebreton M, et al. Knowledge, attitude and practices of snakebite management amongst health workers in Cameroon: Need for continuous training and capacity building. PLoS Negl Trop Dis. 2018;12: e0006716. doi: 10.1371/journal.pntd.0006716 30359385 PMC6219812

[18] Aron MB, Kachimanga C, Kreuels B, Mailosi B, Sambani C, Matanje BL, et al. Health care workers’ knowledge on identification, management and treatment of snakebite cases in rural Malawi: A descriptive study. PLoS Negl Trop Dis. 2022;16: e0010841. doi: 10.1371/journal.pntd.0010841 36409666 PMC9678285

[19] Malik A, Chatterjee K. Awareness of Indian medical practitioners about snakebite and its management—Is there a need to re-evaluate medical training? Med J Dr DY Patil Vidyapeeth. 2020;13: 519–524. doi: 10.4103/mjdrdypu.mjdrdypu_256_19

[20] Visser L., Kyei-Faried S, Belcher D. Protocol and monitoring to improve snake bite outcomes in rural Ghana. Trans R Soc Trop Med Hyg. 2004;98: 278–283. doi: 10.1016/S0035-9203(03)00065-8 15109550

[21] World Health Organization (WHO). Snakebite envenoming- A strategy for prevention and control. Geneva; 2019 [cited 2024 Nov 17]. Available from: https://www.who.int/publications/i/item/9789241515641

[22] The World Bank. Population, total—Eswatini. 2023 [cited 2023 Nov 21]. Available: https://data.worldbank.org/indicator/SP.POP.TOTL?locations=SZ

[23] The World Bank. Data: Eswatini. 2023 [cited 2023 Nov 23]. Available from: https://data.worldbank.org/country/Eswatini

[24] Central Intelligence Agency. Eswatini: Labor force- by occupation. In: The World Factbook. 2023 [cited 2023 Nov 23]. Available from: https://www.cia.gov/the-world-factbook/countries/eswatini/

[25] Food and Agriculture Organization of the United Nations. Swaziland looks to revitalized agricultural sector. 16 Jun 2016 [cited 2023 Nov 23]. Available from: https://www.fao.org/in-action/swaziland-looks-to-a-revitalized-agriculture-sector/en/

[26] Ministry of Health- Kingdom of Eswatini. National Snakebite Management Guidelines. 2021 [cited 2024 Nov 17]. Available from: https://eswatiniantivenom.org/newsite/wp-content/uploads/2021/12/WHO-Snake-Bite-Treatment-Protocol-Manuscript-A4.pdf

[27] Eswatini Antivenom Foundation. Training. In: All Our Training Courses. [cited 2023 Nov 23]. Available from: https://eswatiniantivenom.org/training/

[28] Hicks C, Hennessy D. Hennessy-Hicks Training Needs Analysis Questionnaire and Manual. 2011 [cited 2024 Nov 17]. Available from: http://epapers.bham.ac.uk/3453/10/HenneseyToolkit.pdf

[29] Woodcock T, Adeleke Y, Goeschel C, Pronovost P, Dixon-Woods M. A modified Delphi study to identify the features of high quality measurement plans for healthcare improvement projects. BMC Med Res Methodol. 2020;20. doi: 10.1186/s12874-019-0886-6 31937262 PMC6961316

[30] Jünger S, Payne SA, Brine J, Radbruch L, Brearley SG. Guidance on Conducting and REporting DElphi Studies (CREDES) in palliative care: Recommendations based on a methodological systematic review. Palliat Med. 2017;3: 684–706. doi: 10.1177/0269216317690685 28190381

[31] Runfola D, Anderson A, Baier H, Crittenden M, Dowker E, Fuhrig S, et al. GeoBoundaries: A global database of political administrative boundaries. PLoS One. 2020;15: e0231866. doi: 10.1371/journal.pone.0231866 32330167 PMC7182183

[32] REDCap. REDCap Mobile Device Applications. [cited 2022 Jul 19]. Available from: https://projectredcap.org/software/mobile-app/

[33] Zoom Video Communications, Inc. San Jose, CA, USA; 2021 [cited 2024 Nov 17]. Available from: https://zoom.us

[34] Bärnighausen T, Bloom DE, Humair S. Going Horizontal—Shifts in Funding of Global Health Interventions. N Engl J Med. 2011;364: 2181–2183. doi: 10.1056/NEJMp1014255 21651390

[35] Eswatini. Health Labour Market Analysis for Eswatini. 2023 [cited 2024 Nov 17]. Available from: https://files.aho.afro.who.int/afahobckpcontainer/production/files/Final_HLMA_Report_Eswatini_Signed_and_Launched.pdf

[36] Eswatini Antivenom Foundation. All Our Training Courses. [cited 2024 Feb 23]. Available from: https://eswatiniantivenom.org/training/

[37] The World Health Organization. Health researchers (in full-time equivalent) per million inhabitants, by WHO Region. 2023 [cited 2024 Feb 23]. Available from: https://www.who.int/observatories/global-observatory-on-health-research-and-development/indicators/health-researchers-in-full-time-equivalent-per-million-inhabitants-by-income-group-second-set-of-charts

[38] TDR- The Special Programme for Research and Training in Tropical Diseases. Tackling snakebites in Kenya using operational research. In: Newsroom. 2020 [cited 2024 Jul 8]. Available from: https://tdr.who.int/newsroom/news/item/09-06-2020-tackling-snakebites-in-kenya-using-operational-research

[39] National Institutes of Health (NIH). Siyakhula: Growing HIV/TB Research Knowledge for Growing Healthy Kids in Eswatini [cited 2024 May 2]. Available from: https://reporter.nih.gov/search/Fp7qJ2LfDUay9291Jx4jsg/project-details/10871957

[40] Oluoch GO, Stienstra Y, Schurer JM, Mijumbi R, Mbonigaba JB, Thomas B, et al. Correspondence: The African snakebite Alliance. Toxicon. 2024;237. doi: 10.1016/j.toxicon.2023.107535 38040061

[41] Afroz A, Siddiquea BN, Shetty AN, Jackson TNW, Watt AD. Assessing knowledge and awareness regarding snakebite and management of snakebite envenoming in healthcare workers and the general population: A systematic review and meta-analysis. PLoS Negl Trop Dis. 2023;17: e0011048. doi: 10.1371/journal.pntd.0011048 36757933 PMC9910687

[42] Bala AA, Jatau AI, Yunusa I, Mohammed M, Mohammed AKH, Isa AM, et al. Knowledge assessment of anti-snake venom among healthcare practitioners in northern Nigeria. Ther Adv Infect Dis. 2021;8. doi: 10.1177/20499361211039379 34434552 PMC8381460

[43] Ameade EPK, Bonney I, Boateng ET. Health professionals’ overestimation of knowledge on snakebite management, a threat to the survival of snakebite victims—a cross-sectional study in Ghana. PLoS Negl Trop Dis. 2021;15: e0008756. doi: 10.1371/journal.pntd.0008756 33465084 PMC7846110

[44] Wafula ST, Mugume IB, Namakula LN, Nalugya A, Naggayi V, Walekhwa AW, et al. Healthcare practitioners’ knowledge of snakebite management and associated factors in high-burden, low-resource settings in Uganda. Trans R Soc Trop Med Hyg. 2023;117: 569–579. doi: 10.1093/trstmh/trad015 37072287

[45] Shahmy S, Kularatne SAM, Gawarammana IB, Rathnayake SS, Dawson AH. Compliance with national snakebite treatment guidelines in rural Sri Lankan hospitals: a cluster randomized controlled trial of a brief educational intervention. BMC Med Educ. 2023;23: 390. doi: 10.1186/s12909-023-04375-1 37245040 PMC10225084

[46] Momanyi GO, Adoyo MA, Mwangi EM, Mokua DO. Value of training on motivation among health workers in Narok county, Kenya. Pan Afr Med J. 2016;23: 261. doi: 10.11604/pamj.2016.23.261.8414 27516826 PMC4963175

[47] Bartlett KR, Kang DS. Training and organizational commitment among nurses following industry and organizational change in New Zealand and the United States. Hum Resour Dev Int. 2004;7: 423–440. doi: 10.1080/1367886042000299799

[48] Wafula ST, Namakula LN, Ninsiima LR, Ssekamatte NK, Walekhwa AW, Mugume IB, et al. Barriers and opportunities for improving management of snakebites: Perspectives of healthcare workers in Northern Uganda. PLoS One. 2023;18: e0291032. doi: 10.1371/journal.pone.0291032 37747844 PMC10519583

[49] Rocha G dos S, Souza Rodrigues MF, Rocha YV, Beckman de Lima H, Ramos FR, Teixeira E, et al. Perceptions of nurses regarding the management of snakebite envenomations: Limits and possibilities. Toxicon. 2023;223. doi: 10.1016/j.toxicon.2022.106995 36566992

[50] Barnes K, Ngari C, Parkurito S, Wood L, Otundo D, Harrison R, et al. Delays, fears and training needs: Perspectives of health workers on clinical management of snakebite revealed by a qualitative study in Kitui County, Kenya. Toxicon X. 2021;11. doi: 10.1016/j.toxcx.2021.100078 34401745 PMC8350493

[51] Tankwanchi ABS, Özden Ç, Vermund SH. Physician Emigration from Sub-Saharan Africa to the United States: Analysis of the 2011 AMA Physician Masterfile. PLoS Med. 2013;10: e1001513. doi: 10.1371/journal.pmed.1001513 24068894 PMC3775724

[52] Kruger J, Dunning D. Unskilled and unaware of it: How difficulties in recognizing one’s own incompetence lead to inflated self-assessments. J Pers Soc Psychol. 1999;77: 1121–1134. doi: 10.1037//0022-3514.77.6.1121 10626367

[53] Ooms GI, van Oirschot J, Waldmann B, von Bernus S, van den Ham HA, Mantel-Teeuwisse AK, et al. The current state of snakebite care in Kenya, Uganda, and Zambia: Healthcare workers’ perspectives and knowledge, and health facilities’ treatment capacity. Am J Trop Med Hyg. 2021;104: 774–782. doi: 10.4269/ajtmh.20-1078 33236717 PMC7866361

[54] Salm-Reifferscheidt L. Thea Litschka-Koen: the snake lady of eSwatini. Lancet. 2020;395: 19–20. doi: 10.1016/S0140-6736(19)33169-1 31929003

[55] The World Health Organization. Promoting the integrated approach to skin-related neglected tropical diseases. In: Activities. 2024 [cited 2024 Jul 8]. Available: https://www.who.int/activities/promoting-the-integrated-approach-to-skin-related-neglected-tropical-diseases

[56] Keeney S, Hasson F, Mckenna H. The Delphi Technique in Nursing and Health Research. Chichester, United Kingdom: John Wiley & Sons Ltd; 2011. doi: 10.1002/9781444392029

[57] Adewole DA, Salawu, Mobolaji M, Bello S. Training needs assessment and preferred approach to enhancing work performance among clinical nurses in University College Hospital (UCH), Ibadan, Oyo State, South-western Nigeria. Int J Nurs Midwifery. 2020;12: 130–138. doi: 10.5897/ijnm2020.0434

[58] Holloway K, Arcus K, Orsborn G. Training needs analysis–The essential first step for continuing professional development design. Nurse Educ Pract. 2018;28: 7–12. doi: 10.1016/j.nepr.2017.09.001 28926754

[59] Johnson CF, Earle-Payne K. Identifying mental health training needs of general practice pharmacy workforce to advance practice: a training needs analysis survey. Int J Clin Pharm. 2022;44: 1454–1463. doi: 10.1007/s11096-022-01486-5 36282414

