# Supplementary material for: Developing and applying a training needs analysis tool for healthcare workers managing snakebite envenoming: A cross-sectional study in Eswatini
Source: PLoS Negl Trop Dis. 2025 Jan 8;19(1):e0012778. doi: 10.1371/journal.pntd.0012778 (PMC11709266; doi:10.1371/journal.pntd.0012778)
Supplement: S1 Appendix — (PDF) [file pntd.0012778.s001.pdf]

S1 Appendix. Checklist for Conducting and Reporting Delphi Studies in palliative care (CREDES)

		**Page location in the main document**
**Rationale for the choice of the Delphi technique**	Justification	9
**Planning and design**	Planning and process	6, 7, 8, 9
	Definition of consensus	9
**Study conduct**	Informational input	8, 9
	Prevention of bias	14
	Interpretation and processing of results	13, 14
	External validation	Not performed
**Reporting**	Purpose and rationale	6, 7, 8, 9
	Expert panel	9, 13
	Description of the methods	6, 7, 8, 9
	Procedure	Not performed
	Definition and attainment of consensus	9, 13, 14
	Results	13, 14
	Discussion of limitations	29
	Adequacy of conclusions	25, 29
	Publication and dissemination	In supplementary material
