# Supplementary material for: Developing and applying a training needs analysis tool for healthcare workers managing snakebite envenoming: A cross-sectional study in Eswatini
Source: PLoS Negl Trop Dis. 2025 Jan 8;19(1):e0012778. doi: 10.1371/journal.pntd.0012778 (PMC11709266; doi:10.1371/journal.pntd.0012778)
Supplement: S2 Appendix — (PDF) [file pntd.0012778.s002.pdf]

**S2 Appendix. SB-TNA Tool Delphi Panel-approved version**

# Clinical Training Needs Assessment Eswatini

Record ID ______________________________

## SECTION 1: BACKGROUND INFORMATION

Please choose the name of the hospital/facility you work in. The name of your hospital/facility will not be used when analysing the data you enter in this survey.

- ◯ Good Shepherd Mission Hospital
- ◯ Raleigh Fitkin Memorial Hospital
- ◯ Mankayane Government Hospital
- ◯ Piggs Peak Government Hospital
- ◯ Mbabane Government Hospital
- ◯ Hlatikhulu Government Hospital
- ◯ The Luke Commission Hospital
- ◯ Matsanjeni Health Centre
- ◯ Dvokolwako Health Centre
- ◯ Emkhuzweni Health Centre
- ◯ Sithobela Rural Health Centre
- ◯ Nhlangani Health Centre
- ◯ Mhlume RES Clinic
- ◯ Simunye RES Clinic
- ◯ Ngomane RES Clinic

What type of facility do you work in?

- ◯ Clinic
- ◯ Hospital
- ◯ Health centre
- ◯ National referral hospital
- ◯ Other

Please specify in what facility/ what capacity you care for patients. ______________________________

Please indicate your sex (male/female) here.

- ◯ Female
- ◯ Male

Please indicate how old you are (in years).

- ◯ 20-29
- ◯ 30-39
- ◯ 40-49
- ◯ 50-59
- ◯ 60+

What is your professional role?

- ◯ Nursing aide
- ◯ Registered nurse
- ◯ Nurse assistant
- ◯ Matron
- ◯ Medical officer
- ◯ Pharmacist
- ◯ Specialist
- ◯ Paramedic
- ◯ Medical doctor
- ◯ Other

Please specify/ describe your professional role. ______________________________

What is your job title? ______________________________

| | |
|---|---|
| What is your primary qualification? | ◯ Nursing Certificate<br>◯ Medical Degree<br>◯ Nursing Diploma<br>◯ Nursing Degree<br>◯ Pharmacist<br>◯ Paramedic training<br>◯ Other |
| Please specify your primary qualification. | ________________________ |
| How many years ago did you achieve your primary qualification? | ________________________ |
| Which department do you work in? | ☐ General Outpatient<br>☐ Surgery<br>☐ Accident & Emergency<br>☐ Internal Medicine<br>☐ Paediatrics<br>☐ Ambulance<br>☐ Maternity<br>☐ Public health unit<br>☐ Other |
| Please specify the department you work in. | ________________________ |
| How many cases of snakebite envenoming have you been involved with at work in the last 2 years? | ◯ 0<br>◯ 1-2<br>◯ 3-9<br>◯ 10-20<br>◯ >20 |
| Did you receive any training on snakebite management as part of your primary healthcare qualification, e.g. nursing diploma/medical degree? | ◯ Yes<br>◯ No |
| If yes, estimate how many hours of snakebite training were included in your course. | ________________________ |
| Have you received any training on snakebite management since completing your primary qualification? | ◯ Yes<br>◯ No |
| What programme was this training part of? | ◯ Part of a postgraduate degree<br>◯ Part of a postgraduate diploma<br>◯ A certified training course<br>◯ Continuous medical education/ seminar/ lecture/ symposium<br>◯ Other |
| Please specify what programme this training was part of. | ________________________ |
| Can you provide the name of the training institution or agency? | ☐ The hospital/ facility where I work/ worked<br>☐ The Eswatini Antivenom Foundation<br>☐ A university<br>☐ Other |

| | |
|---|---|
| Can you provide the name of the training institution or agency? | ______________________ |
| What aspects of snakebite were covered as part of this training/ education? | ☐ Snakes, snake venoms and syndromes of envenoming<br>☐ Prevention of snakebite<br>☐ Diagnosis of snakebite<br>☐ Treatment of snakebite<br>☐ Managing adverse antivenin reactions |
| How many hours of snakebite management training have you received since completing your primary qualification? Please estimate. | ______________________ |
| Where, if at all, do you get guidance or support when managing a patient with snakebite? | ☐ Ask peers in your place of work<br>☐ Ask senior staff in your place of work<br>☐ Consult staff in another facility<br>☐ Consult specialist snakebite team<br>☐ Clinical/official guidelines for the management of snakebite<br>☐ Textbook<br>☐ Internet<br>☐ Other<br>☐ I do not receive guidance from anywhere/anyone |
| Please specify what other sources you use to inform yourself about snakebite management. | ______________________ |
| Can you specify the job title/position/profession of the staff member whom you consult for guidance? | ______________________ |
| Can you provide a name of the guideline, textbook and/or website that you use? | ______________________ |

SECTION 2: PERSONAL TRAINING NEEDS

Guidance for completing the Training Needs Assessment Questionnaire

A range of different skills are needed to work in healthcare and to care for patients with snakebite envenoming. To perform these skills effectively, you need training in the relevant skills and a work environment that allows you to exercise your skills appropriately. This questionnaire aims to identify skills that are important for you personally to be able to manage snakebite patients within your job role and whose performance could be best improved by training.

Please answer the questions as honestly as you can as this will help us understand best what is needed to help health workers in Eswatini manage snakebite patients effectively. Your responses are completely confidential. Your responses will not be shared with your supervisor. All returned forms will be anonymised so the information cannot be traced back to an individual.

Further below is a list of skills that may be needed by different healthcare workers who manage snakebite patients. Some of these skills you will use only with patients with snakebite, some with patients with many different conditions and some in your role in general. Please answer every question as it relates to you caring for patients with snakebite. Give your initial responses; a long time reviewing your answers is not required.

For each skill, please indicate how important you think that skill is when caring for patients with suspected snakebite and how well you currently perform that skill.

'Importance'

1 2 3 4 5 6 7

'Own performance'

1 2 3 4 5 6 7

For illustration, the example rating below shows a skill you think is a bit important (score= 5), but not well performed (score= 2).

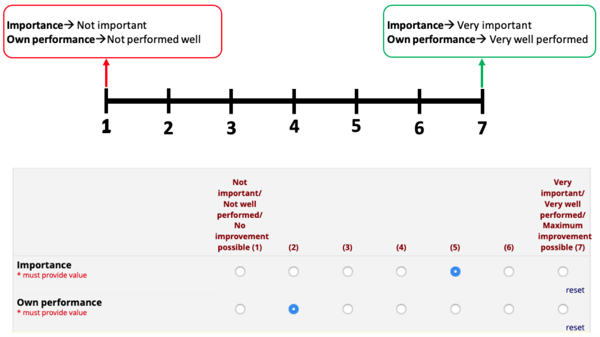

**Appropriate management of mildly or non-envenomed snakebite patients**

| | Not important/ Not well performed/ No improvement possible (1) | (2) | (3) | (4) | (5) | (6) | Very important/ Very well performed/ Maximum improvement possible (7) |
|---|---|---|---|---|---|---|---|
| Importance | ○ | ○ | ○ | ○ | ○ | ○ | ○ |
| Own performance | ○ | ○ | ○ | ○ | ○ | ○ | ○ |

**Prioritising tasks to maintain effective care when dealing with competing patient demands or limited staffing**

| | Not important/ Not well performed/ No improvement possible (1) | (2) | (3) | (4) | (5) | (6) | Very important/ Very well performed/ Maximum improvement possible (7) |
|---|---|---|---|---|---|---|---|
| Importance | ○ | ○ | ○ | ○ | ○ | ○ | ○ |
| Own performance | ○ | ○ | ○ | ○ | ○ | ○ | ○ |

**Assess and treat venom ophthalmia**

| | Not important/ Not well performed/ No improvement possible (1) | (2) | (3) | (4) | (5) | (6) | Very important/ Very well performed/ Maximum improvement possible (7) |
|---|---|---|---|---|---|---|---|
| Importance | ○ | ○ | ○ | ○ | ○ | ○ | ○ |
| Own performance | ○ | ○ | ○ | ○ | ○ | ○ | ○ |

**Reviewing clinical care in your facility and identifying areas suitable for research, audit or quality improvement**

| | Not important/ Not well performed/ No improvement possible (1) | (2) | (3) | (4) | (5) | (6) | Very important/ Very well performed/ Maximum improvement possible (7) |
|---|---|---|---|---|---|---|---|

| | | | | | | | |
|---|---|---|---|---|---|---|---|
| Importance | ○ | ○ | ○ | ○ | ○ | ○ | ○ |
| Own performance | ○ | ○ | ○ | ○ | ○ | ○ | ○ |

**Accurately diagnosing snakebite envenoming and identifying the syndrome of envenoming**

| | Not important/ Not well performed/ No improvement possible (1) | (2) | (3) | (4) | (5) | (6) | Very important/ Very well performed/ Maximum improvement possible (7) |
|---|---|---|---|---|---|---|---|
| Importance | ○ | ○ | ○ | ○ | ○ | ○ | ○ |
| Own performance | ○ | ○ | ○ | ○ | ○ | ○ | ○ |

**Understand the indications for and choice of appropriate antibiotics in snakebite-associated skin and soft tissue infections**

| | Not important/ Not well performed/ No improvement possible (1) | (2) | (3) | (4) | (5) | (6) | Very important/ Very well performed/ Maximum improvement possible (7) |
|---|---|---|---|---|---|---|---|
| Importance | ○ | ○ | ○ | ○ | ○ | ○ | ○ |
| Own performance | ○ | ○ | ○ | ○ | ○ | ○ | ○ |

**Advocating for, participating in and applying learning from quality improvement initiatives**

| | Not important/ Not well performed/ No improvement possible (1) | (2) | (3) | (4) | (5) | (6) | Very important/ Very well performed/ Maximum improvement possible (7) |
|---|---|---|---|---|---|---|---|
| Importance | ○ | ○ | ○ | ○ | ○ | ○ | ○ |
| Own performance | ○ | ○ | ○ | ○ | ○ | ○ | ○ |

## Appraising your own performance

| | Not important/ Not well performed/ No improvement possible (1) | (2) | (3) | (4) | (5) | (6) | Very important/ Very well performed/ Maximum improvement possible (7) |
|---|---|---|---|---|---|---|---|
| Importance | ○ | ○ | ○ | ○ | ○ | ○ | ○ |
| Own performance | ○ | ○ | ○ | ○ | ○ | ○ | ○ |

## Confident in assessing wounds and providing wound care for local tissue damage, including dressings

| | Not important/ Not well performed/ No improvement possible (1) | (2) | (3) | (4) | (5) | (6) | Very important/ Very well performed/ Maximum improvement possible (7) |
|---|---|---|---|---|---|---|---|
| Importance | ○ | ○ | ○ | ○ | ○ | ○ | ○ |
| Own performance | ○ | ○ | ○ | ○ | ○ | ○ | ○ |

## Knowing the indications for tissue debridement and skin grafting

| | Not important/ Not well performed/ No improvement possible (1) | (2) | (3) | (4) | (5) | (6) | Very important/ Very well performed/ Maximum improvement possible (7) |
|---|---|---|---|---|---|---|---|
| Importance | ○ | ○ | ○ | ○ | ○ | ○ | ○ |
| Own performance | ○ | ○ | ○ | ○ | ○ | ○ | ○ |

## Identifying and managing adverse effects of antivenom, including anaphylaxis

| | Not important/ Not well performed/ No improvement possible (1) | (2) | (3) | (4) | (5) | (6) | Very important/ Very well performed/ Maximum improvement possible (7) |
|---|---|---|---|---|---|---|---|

| | | | | | | | |
|---|---|---|---|---|---|---|---|
| Importance | ◯ | ◯ | ◯ | ◯ | ◯ | ◯ | ◯ |
| Own performance | ◯ | ◯ | ◯ | ◯ | ◯ | ◯ | ◯ |

**Understanding specific considerations when managing snakebite envenoming in children, pregnant women and patients with severe co-morbidities**

| | Not important/ Not well performed/ No improveme nt possible (1) | (2) | (3) | (4) | (5) | (6) | Very important/ Very well performed/ Maximum improveme nt possible (7) |
|---|---|---|---|---|---|---|---|
| Importance | ◯ | ◯ | ◯ | ◯ | ◯ | ◯ | ◯ |
| Own performance | ◯ | ◯ | ◯ | ◯ | ◯ | ◯ | ◯ |

**Aware of the risks of fasciotomy and rare scenarios in which it might be indicated**

| | Not important/ Not well performed/ No improveme nt possible (1) | (2) | (3) | (4) | (5) | (6) | Very important/ Very well performed/ Maximum improveme nt possible (7) |
|---|---|---|---|---|---|---|---|
| Importance | ◯ | ◯ | ◯ | ◯ | ◯ | ◯ | ◯ |
| Own performance | ◯ | ◯ | ◯ | ◯ | ◯ | ◯ | ◯ |

**Recognizing and understanding the importance of hydration status, the need to record, monitor and assess fluid balance and take into account relevant laboratory findings**

| | Not important/ Not well performed/ No improveme nt possible (1) | (2) | (3) | (4) | (5) | (6) | Very important/ Very well performed/ Maximum improveme nt possible (7) |
|---|---|---|---|---|---|---|---|
| Importance | ◯ | ◯ | ◯ | ◯ | ◯ | ◯ | ◯ |
| Own performance | ◯ | ◯ | ◯ | ◯ | ◯ | ◯ | ◯ |

**Knowing the indications for antivenom treatment and which antivenom to administer for different envenoming syndromes in your setting**

| | Not important/ Not well performed/ No improvement possible (1) | (2) | (3) | (4) | (5) | (6) | Very important/ Very well performed/ Maximum improvement possible (7) |
|---|---|---|---|---|---|---|---|
| Importance | ○ | ○ | ○ | ○ | ○ | ○ | ○ |
| Own performance | ○ | ○ | ○ | ○ | ○ | ○ | ○ |

**Contributing to and engaging in continuing medical education**

| | Not important/ Not well performed/ No improvement possible (1) | (2) | (3) | (4) | (5) | (6) | Very important/ Very well performed/ Maximum improvement possible (7) |
|---|---|---|---|---|---|---|---|
| Importance | ○ | ○ | ○ | ○ | ○ | ○ | ○ |
| Own performance | ○ | ○ | ○ | ○ | ○ | ○ | ○ |

**Including information/messages on snakebite in community engagement and health promotion**

| | Not important/ Not well performed/ No improvement possible (1) | (2) | (3) | (4) | (5) | (6) | Very important/ Very well performed/ Maximum improvement possible (7) |
|---|---|---|---|---|---|---|---|
| Importance | ○ | ○ | ○ | ○ | ○ | ○ | ○ |
| Own performance | ○ | ○ | ○ | ○ | ○ | ○ | ○ |

**Giving information about snakebite care to patients and/or carers**

| | Not important/ Not well performed/ No improvement possible (1) | (2) | (3) | (4) | (5) | (6) | Very important/ Very well performed/ Maximum improvement possible (7) |
|---|---|---|---|---|---|---|---|

| | | | | | | | |
|---|---|---|---|---|---|---|---|
| Importance | ○ | ○ | ○ | ○ | ○ | ○ | ○ |
| Own performance | ○ | ○ | ○ | ○ | ○ | ○ | ○ |

**Personally coping with stressors in health care, such as challenging cases, deaths and changing or difficult working conditions**

| | Not important/ Not well performed/ No improvement possible (1) | (2) | (3) | (4) | (5) | (6) | Very important/ Very well performed/ Maximum improvement possible (7) |
|---|---|---|---|---|---|---|---|
| Importance | ○ | ○ | ○ | ○ | ○ | ○ | ○ |
| Own performance | ○ | ○ | ○ | ○ | ○ | ○ | ○ |

**Being aware of the medically important venomous snakes in your area and the syndromes they cause**

| | Not important/ Not well performed/ No improvement possible (1) | (2) | (3) | (4) | (5) | (6) | Very important/ Very well performed/ Maximum improvement possible (7) |
|---|---|---|---|---|---|---|---|
| Importance | ○ | ○ | ○ | ○ | ○ | ○ | ○ |
| Own performance | ○ | ○ | ○ | ○ | ○ | ○ | ○ |

**Providing advice to patients and/or carers on snakebite prevention, appropriate community first aid for snakebite and care seeking**

| | Not important/ Not well performed/ No improvement possible (1) | (2) | (3) | (4) | (5) | (6) | Very important/ Very well performed/ Maximum improvement possible (7) |
|---|---|---|---|---|---|---|---|
| Importance | ○ | ○ | ○ | ○ | ○ | ○ | ○ |
| Own performance | ○ | ○ | ○ | ○ | ○ | ○ | ○ |

## Establishing a relationship with patients and/or carers

| | Not important/ Not well performed/ No improvement possible (1) | (2) | (3) | (4) | (5) | (6) | Very important/ Very well performed/ Maximum improvement possible (7) |
|---|---|---|---|---|---|---|---|
| Importance | ○ | ○ | ○ | ○ | ○ | ○ | ○ |
| Own performance | ○ | ○ | ○ | ○ | ○ | ○ | ○ |

## Identifying life- or limb-threatening conditions and managing them appropriately using an ABCDE approach

| | Not important/ Not well performed/ No improvement possible (1) | (2) | (3) | (4) | (5) | (6) | Very important/ Very well performed/ Maximum improvement possible (7) |
|---|---|---|---|---|---|---|---|
| Importance | ○ | ○ | ○ | ○ | ○ | ○ | ○ |
| Own performance | ○ | ○ | ○ | ○ | ○ | ○ | ○ |

## Performing and interpreting relevant available point-of-care and/or laboratory tests, e.g. the 20 Minute Whole Blood Clotting Test

| | Not important/ Not well performed/ No improvement possible (1) | (2) | (3) | (4) | (5) | (6) | Very important/ Very well performed/ Maximum improvement possible (7) |
|---|---|---|---|---|---|---|---|
| Importance | ○ | ○ | ○ | ○ | ○ | ○ | ○ |
| Own performance | ○ | ○ | ○ | ○ | ○ | ○ | ○ |

## Assess and manage pain using appropriate analgesics

| | Not important/ Not well performed/ No improvement possible (1) | (2) | (3) | (4) | (5) | (6) | Very important/ Very well performed/ Maximum improvement possible (7) |
|---|---|---|---|---|---|---|---|

| | | | | | | | |
|---|---|---|---|---|---|---|---|
| Importance | ○ | ○ | ○ | ○ | ○ | ○ | ○ |
| Own performance | ○ | ○ | ○ | ○ | ○ | ○ | ○ |

**Accessing and applying guidance and other information on snakebite management**

| | Not important/ Not well performed/ No improveme nt possible (1) | (2) | (3) | (4) | (5) | (6) | Very important/ Very well performed/ Maximum improveme nt possible (7) |
|---|---|---|---|---|---|---|---|
| Importance | ○ | ○ | ○ | ○ | ○ | ○ | ○ |
| Own performance | ○ | ○ | ○ | ○ | ○ | ○ | ○ |

**Writing accurate, timely and good quality clinical records and referral letters**

| | Not important/ Not well performed/ No improveme nt possible (1) | (2) | (3) | (4) | (5) | (6) | Very important/ Very well performed/ Maximum improveme nt possible (7 |
|---|---|---|---|---|---|---|---|
| Importance | ○ | ○ | ○ | ○ | ○ | ○ | ○ |
| Own performance | ○ | ○ | ○ | ○ | ○ | ○ | ○ |

**Confident in your role in facility preparedness to deliver effective care, including ensuring relevant equipment, resources and supplies are available in a timely manner**

| | Not important/ Not well performed/ No improveme nt possible (1) | (2) | (3) | (4) | (5) | (6) | Very important/ Very well performed/ Maximum improveme nt possible (7) |
|---|---|---|---|---|---|---|---|
| Importance | ○ | ○ | ○ | ○ | ○ | ○ | ○ |
| Own performance | ○ | ○ | ○ | ○ | ○ | ○ | ○ |

**Collecting and collating relevant information for audit, research or a disease register**

| | Not important/ Not well performed/ No improvement possible (1) | (2) | (3) | (4) | (5) | (6) | Very important/ Very well performed/ Maximum improvement possible (7) |
|---|---|---|---|---|---|---|---|
| Importance | ○ | ○ | ○ | ○ | ○ | ○ | ○ |
| Own performance | ○ | ○ | ○ | ○ | ○ | ○ | ○ |

**Administering antivenom treatment at an appropriate dose, rate and route, with pre-medication when appropriate**

| | Not important/ Not well performed/ No improvement possible (1) | (2) | (3) | (4) | (5) | (6) | Very important/ Very well performed/ Maximum improvement possible (7) |
|---|---|---|---|---|---|---|---|
| Importance | ○ | ○ | ○ | ○ | ○ | ○ | ○ |
| Own performance | ○ | ○ | ○ | ○ | ○ | ○ | ○ |

**Able to assess a patient with neurotoxicity, safely manage an airway and recognise when assisted ventilation is needed**

| | Not important/ Not well performed/ No improvement possible (1) | (2) | (3) | (4) | (5) | (6) | Very important/ Very well performed/ Maximum improvement possible (7) |
|---|---|---|---|---|---|---|---|
| Importance | ○ | ○ | ○ | ○ | ○ | ○ | ○ |
| Own performance | ○ | ○ | ○ | ○ | ○ | ○ | ○ |

**Working as a member of a team**

| | Not important/ Not well performed/ No improvement possible (1) | (2) | (3) | (4) | (5) | (6) | Very important/ Very well performed/ Maximum improvement possible (7) |
|---|---|---|---|---|---|---|---|

| | | | | | | | |
|---|---|---|---|---|---|---|---|
| Importance | ◯ | ◯ | ◯ | ◯ | ◯ | ◯ | ◯ |
| Own performance | ◯ | ◯ | ◯ | ◯ | ◯ | ◯ | ◯ |

**Able to recognise clinical signs of shock and initiate appropriate treatment when indicated**

| | Not important/ Not well performed/ No improvement possible (1) | (2) | (3) | (4) | (5) | (6) | Very important/ Very well performed/ Maximum improvement possible (7) |
|---|---|---|---|---|---|---|---|
| Importance | ◯ | ◯ | ◯ | ◯ | ◯ | ◯ | ◯ |
| Own performance | ◯ | ◯ | ◯ | ◯ | ◯ | ◯ | ◯ |

**Able to support ventilation when indicated using appropriate and available resources**

| | Not important/ Not well performed/ No improvement possible (1) | (2) | (3) | (4) | (5) | (6) | Very important/ Very well performed/ Maximum improvement possible (7) |
|---|---|---|---|---|---|---|---|
| Importance | ◯ | ◯ | ◯ | ◯ | ◯ | ◯ | ◯ |
| Own performance | ◯ | ◯ | ◯ | ◯ | ◯ | ◯ | ◯ |

**Making appropriate monitoring and discharge or referral plans for patients with snakebite envenoming**

| | Not important/ Not well performed/ No improvement possible (1) | (2) | (3) | (4) | (5) | (6) | Very important/ Very well performed/ Maximum improvement possible (7) |
|---|---|---|---|---|---|---|---|
| Importance | ◯ | ◯ | ◯ | ◯ | ◯ | ◯ | ◯ |
| Own performance | ◯ | ◯ | ◯ | ◯ | ◯ | ◯ | ◯ |

## Making best use of limited resources within your facility and geographical area

| | Not important/ Not well performed/ No improvement possible (1) | (2) | (3) | (4) | (5) | (6) | Very important/ Very well performed/ Maximum improvement possible (7) |
|---|---|---|---|---|---|---|---|
| Importance | ○ | ○ | ○ | ○ | ○ | ○ | ○ |
| Own performance | ○ | ○ | ○ | ○ | ○ | ○ | ○ |

## Assessment of patient with suspected snakebite, including history and examination

| | Not important/ Not well performed/ No improvement possible (1) | (2) | (3) | (4) | (5) | (6) | Very important/ Very well performed/ Maximum improvement possible (7) |
|---|---|---|---|---|---|---|---|
| Importance | ○ | ○ | ○ | ○ | ○ | ○ | ○ |
| Own performance | ○ | ○ | ○ | ○ | ○ | ○ | ○ |

## Effective communication with your colleagues

| | Not important/ Not well performed/ No improvement possible (1) | (2) | (3) | (4) | (5) | (6) | Very important/ Very well performed/ Maximum improvement possible (7) |
|---|---|---|---|---|---|---|---|
| Importance | ○ | ○ | ○ | ○ | ○ | ○ | ○ |
| Own performance | ○ | ○ | ○ | ○ | ○ | ○ | ○ |

## Recognising the potential psychological and social impact of snakebite envenoming and being aware of available support services

| | Not important/ Not well performed/ No improvement possible (1) | (2) | (3) | (4) | (5) | (6) | Very important/ Very well performed/ Maximum improvement possible (7) |
|---|---|---|---|---|---|---|---|

| | | | | | | | |
|---|---|---|---|---|---|---|---|
| Importance | ◯ | ◯ | ◯ | ◯ | ◯ | ◯ | ◯ |
| Own performance | ◯ | ◯ | ◯ | ◯ | ◯ | ◯ | ◯ |

**Assessing clinical response to antivenom and determining when additional doses are required**

| | Not important/ Not well performed/ No improvement possible (1) | (2) | (3) | (4) | (5) | (6) | Very important/ Very well performed/ Maximum improvement possible (7) |
|---|---|---|---|---|---|---|---|
| Importance | ◯ | ◯ | ◯ | ◯ | ◯ | ◯ | ◯ |
| Own performance | ◯ | ◯ | ◯ | ◯ | ◯ | ◯ | ◯ |

**SECTION 3: TEAM TRAINING NEEDS**

**Please specify the areas of snakebite management in which you think your team needs further training or instruction. List as many as you like in order of importance. Please list any skills or training needs you think are important, whether or not they were included in the questionnaire that you just completed. Put the area you think is most important in the box labelled 'Point 1', followed by your second, third, and fourth suggestions, etc. in the boxes that follow. You do not need to fill-in every box. In case you have no further suggestions, skip the boxes to complete the survey.**

____________________
(Point 1 to be improved (list the most important thing requiring change here, with those that are less important in descending order below) )

____________________
(Point 2 to be improved)

____________________
(Point 3 to be improved)

____________________
(Point 4 to be improved)

____________________
(Point 5 to be improved)

Notes or Additions
