# Supplementary material for: Developing and applying a training needs analysis tool for healthcare workers managing snakebite envenoming: A cross-sectional study in Eswatini
Source: PLoS Negl Trop Dis. 2025 Jan 8;19(1):e0012778. doi: 10.1371/journal.pntd.0012778 (PMC11709266; doi:10.1371/journal.pntd.0012778)
Supplement: S4 Appendix — (PDF) [file pntd.0012778.s004.pdf]

S4 Appendix. Content of healthcare worker training courses on the management of snakebite envenoming offered by the Eswatini Antivenom Foundation

The training offered to HCWs by the Eswatini Antivenom Foundation is primarily theoretical with an OSCE style demonstration. The following topics are taught as part of the training:

- Snake species, different kinds of venoms, clinical syndromes of envenoming
- First-aid and emergency management of snakebite envenoming
- Antivenom, how it is produced and what it contains
- Indications for antivenom
- Managing snakebites in children with emphasis on antivenom dose requirements (same dose of antivenom to be given to children as to adults)
- How neurotoxic venom works
- Ventilation of snakebite victims
- Venom ophthalmia
- Wound management
- Indications for surgical procedures (e.g. debridement, fasciotomy, compartment syndrome) in SBE
- Introduction to the Eswatini Snakebite Management Guidelines
- A short introduction to research in SBE

Towards the end of the training, a panel discussion is held in which HCWs can ask questions, myths about snakes are debunked and live snakes that are brought along are showcased to increase the awareness of different snake species among HCWs. The training lasts a full day, usually starting at nine o’clock in the morning and lasting until six o’clock in the evening.
